# Buckwheat Husk: An Underexplored Source of Bioactive Compounds and Functional Food Applications

**DOI:** 10.3390/foods15173062

**Published:** 2026-08-29

**Authors:** Wajeeha Mumtaz, Joanna Klepacka, Marta Czarnowska-Kujawska

**Affiliations:** Department of Commodity Science and Food Analysis, Faculty of Food Sciences, University of Warmia and Mazury in Olsztyn, 10-719 Olsztyn, Poland

**Keywords:** buckwheat husk, dietary fiber, phenolic compounds, rutin, techno-functional properties, food by-products

## Abstract

Buckwheat husk is the major by-product produced during buckwheat processing. However, despite its high nutritional and functional value, it remains underutilized as an ingredient in functional foods. This review summarizes current knowledge on the botanical origin, chemical composition, extraction approaches, and bioactive properties of buckwheat husk. Particular emphasis is placed on its high content of insoluble dietary fiber and phenolic compounds, especially rutin, which contribute to antioxidant, anti-inflammatory, and antimicrobial activities. Advances in conventional and green extraction techniques used to obtain buckwheat husk concentrates, including ultrasound- and microwave-assisted methods, are discussed. The techno-functional properties of buckwheat husk, such as water- and oil-holding capacity, emulsifying potential, and texture modification, are evaluated in relation to food applications. Reported uses in bakery products, pasta, dairy foods, beverages, and meat systems are critically reviewed. Safety aspects, allergenicity, and antinutritional factors are also considered. Overall, buckwheat husk is positioned as an underutilized resource with significant potential for sustainable functional food development.

## 1. Introduction

Buckwheat is a dicotyledonous grain crop classified within the genus *Fagopyrum* of the family *Polygonaceae.* It is classified within the cereal plant group owing to the analogous chemical makeup of its seeds, their applications, and agronomic practices [1]. According to the Food and Agriculture Organization (FAO), global buckwheat production reached approximately 2.2 million tons in 2023. Husk by-products constituted 30–40% of the processed material [2,3]. However, the proportion of husk relative to the original grain mass varies depending on the buckwheat species, cultivar, and processing conditions [4]. Moreover, buckwheat is an underutilized crop that presents significant opportunity to enhance variety and establish resilient agro-industrial systems to combat climate change [5]. It is well-suited for cultivation on marginal soils due to its low fertilizer requirements, making it appropriate for low-input agricultural practices. Furthermore, by-products such as husk can be converted into fertilizers, bioenergy, and other value-added products (including food), thereby supporting circular bioeconomy strategies [6].

Buckwheat contains a variety of bioactive compounds, which, in addition to basic nutrients, contribute to positive health benefits. Until now, approximately 180 bioactive compounds have been detected and identified in buckwheat [7]. Buckwheat is recognized as a good source of nutritionally valuable protein, lipid, dietary fiber, and minerals, as well as other health-promoting components, such as phenolic compounds and sterols [8]. Botanically, the buckwheat fruit is an achene consisting of an outer husk (pericarp) surrounding the seed coat, aleurone layer, endosperm, and embryo. The embryo contains two cotyledons that extend through the endosperm toward the outer margins of the seed. Bran is not a distinct botanical structure but a milling fraction containing peripheral seed tissues and portions of the embryo [9]. In the production of buckwheat products such as groats or flour, approximately 17–20% of the total grain mass is removed as husk during dehulling, resulting in a significant amount of by-product [10]. Buckwheat husk contains a greater quantity of beneficial compounds than buckwheat flour [11] and is characterized by a high content of dietary fiber, total polyphenolics, and antioxidant activity [12]. Buckwheat husk contains high levels of insoluble fiber, rutin, protocatechuic acid, and syringaresinol [5]. Its extracts can retard lipid oxidation, while flavonoid glycosides and methylated polyphenols contribute to their antioxidative effects [13]. Buckwheat husk may therefore be used as a valuable ingredient in commonly used bakery products [14,15,16]. Beyond bakery products, buckwheat husk has been tested as a functional ingredient in yogurt, noodles, and tea, while husk extracts have been used in meat products and mayonnaise as natural preservatives to extend shelf life. These studies report that buckwheat husk has been incorporated into yoghurt as a functional additive, added to noodles to enhance dietary fiber content, and utilized in tea preparation. Additionally, husk extracts have been applied in meat products and mayonnaise as natural antioxidants to inhibit lipid oxidation and extend shelf life [17,18,19,20,21].

Buckwheat has received increasing attention as a potential functional food. According to the FUFOSE Consensus Document, a food can be regarded as functional if it beneficially affects one or more target functions in the body beyond adequate nutritional effects, thereby contributing to improved health and well-being and/or a reduced risk of disease [22,23]. Buckwheat is known for its high nutritional value and superior sensory qualities and has the potential to be a part of a healthy diet [24]. Due to its nutritional composition and sensory qualities, buckwheat is traditionally used in the production of various foods [25]. Cooked buckwheat groats (“kasha”) are an important part of traditional cuisine in central and Eastern Europe, including Slovenia, Croatia, Poland, Ukraine, Belarus, and Russia. In Japan, buckwheat is mainly consumed as noodles, soba [26]. Buckwheat flour is unmixed or mixed with wheat flour to prepare bakery products, including bread, blini, and cookies. Buckwheat is also used to make beverages [26,27].

Buckwheat grains undergo dehulling and are subsequently processed into groats and flour for human consumption [28]. The dehulling process produces a substantial quantity of husk as a byproduct of buckwheat processing [29,30]. Dehulled seeds (raw grains subjected to varying degrees of heat treatment) are principally used for human consumption as meal called “kasha”, breakfast cereals or as processed flour for making different bakery products (bread, cookies, snacks, and noodles) enriched with buckwheat flour (0.3−60%) or flours obtained from other cereals, and buckwheat enhanced non-bakery products (tea, honey, tarhana, and sprouts) [31]. Buckwheat is a gluten-free pseudocereal, which may be included in gluten-free diets for patients suffering gluten intolerance [32]. As demonstrated in prior studies, the incorporation of buckwheat flour into baked items enhances their antioxidative potential and sensory properties [33]. The choice of extraction technique plays a critical role in the recovery of bioactive compounds from buckwheat husk. While solvent extraction is still widely employed, emerging methods such as ultrasound-assisted extraction (UAE), microwave-assisted extraction (MAE), pulsed electric field (PEF), and high-pressure processing (HPP) are increasingly being adopted [30].

In recent years, more attention has been paid to using buckwheat by-products, especially buckwheat husk, owing to its high dietary fiber content and substantial levels of bioactive compounds. In the past, buckwheat husk remained insufficiently investigated in both scientific research and commercial applications. Some authors [7,34] discussed buckwheat husk together with sprouts and grain and sprout extracts, but by focusing on other parts of the buckwheat seed or plant, their analysis regarding the buckwheat husk itself remains rather superficial. Our review article therefore isolates the husk as an under-utilized by-product and integrates botanical background, detailed composition, green extraction technologies, techno-functional properties, and safety considerations. This overview aims to identify how this byproduct can be transformed into a high-value functional ingredient for food and nutraceutical applications.

## 2. Literature Review Methodology

A literature search was conducted using Google Scholar, Web of Science, Scopus, PubMed, and ScienceDirect. The main keywords included “buckwheat husk”, “buckwheat hull”, “bioactive compounds”, “phenolic compounds”, “dietary fibre”, “extraction”, “antioxidant activity”, “techno-functional properties”, “food applications”, “safety”, “allergenicity”, and “mycotoxins”, used individually and in appropriate combinations. The search covered literature available to our university in an open system up to August 2026. Studies were included when they provided information relevant to the composition and bioactive compounds, extraction and analytical approaches, biological and techno-functional properties, food applications, or safety of buckwheat husk. Studies unrelated to the scope of the review and duplicate publications were excluded. The selected literature comprised 56 publications addressing composition and bioactive compounds, 37 addressing extraction and analytical approaches, 35 addressing biological and techno-functional properties, 19 addressing food applications, and 20 addressing safety-related aspects. Some publications contributed to more than one of these areas and were therefore represented in more than one category. Following the selection process, 146 unique publications were included in the review. In addition, seven official and regulatory sources were consulted for regulatory and safety information, resulting in a total of 153 references.

## 3. Botanical Characteristics of Buckwheat and Composition of the Husk

### 3.1. Botanical Characteristics and Major Species

Buckwheat is a dicot with irregular and triangular seeds [21]. The seed contains brown-to-black color husk covering the whole kernel, colored white to light green. The color and hardness of buckwheat husk are related to different cultivars of buckwheat [35]. The buckwheat seed germinates in soil during 3–4 days, flowering after 3 weeks of planting with white petals, and after pollination by wind, seed formation starts within 10 days. The buckwheat seed needs more than 1 week to attain full maturity after seed formation and requires low-fertility soil with a moderate nitrogen content [36]. The cultivation period of buckwheat is a short growing period of 70–90 days and good storage periods due to its chemical constituents, especially phenolics.

Buckwheat is a broadleaf pseudocereal that is an annual plant belonging to the family *Polygonaceae*. Buckwheat is related to both sorrel and rhubarb, which makes it a productive crop that can be used for both human use and environmentally responsible agricultural techniques such as crop rotation and the use of cover crops for natural weed suppression and soil improvement [37]. *Fagopyrum tataricum*, also known as Tartary buckwheat, and *Fagopyrum esculentum*, also known as common buckwheat, are the two kinds of buckwheat that are grown the most frequently among the primary varieties of buckwheat that have agricultural value [21,34]. Among the cultivated buckwheat species, *Fagopyrum tataricum* differs from *F. esculentum* in its nutritional and phytochemical profile. Compared with common buckwheat, Tartary buckwheat is characterized by higher levels of protein with a balanced amino acid composition, dietary fiber, vitamins, minerals, and flavonoids, particularly rutin, which occurs at approximately 100-fold higher concentrations than in common buckwheat [38,39]. Both species are characterized by a predominance of unsaturated fatty acids (C18:1, C18:2, C18:3 and C20:1), whereas Tartary buckwheat generally contains higher total levels of B vitamins in both flour and bran fractions [40].

### 3.2. Buckwheat Husk Formation

The initial step in buckwheat processing is decortication, which generates a significant amount of solid waste in the form of buckwheat husk. Despite its nutrient richness, husk is often used as fuel or filling for therapeutic pillows [2]. In industrial practice, harvested buckwheat grains are air-dried and cleaned using winnowing machines to remove chaff and foreign matter [41]. Pre-cleaned buckwheat grains are then hulled mechanically in a sheller, which uses friction and centrifugal force to crack the husk. Grains may also undergo brushing or polishing to remove dust and fungal spores without compromising germination. Traditional dry husking involves grading seeds into fractions before hulling and then separating kernels from shells, whereas wet husking employs hydrothermal conditioning to soften the husk and improve kernel yield. The final products are groats (edible kernels) and by-products like husk, which was used mainly for non-food purposes but is recently also being explored as a source of many nutrients for the food industry [42].

### 3.3. Buckwheat Husk Nutritional Composition and Dietary Fiber

Buckwheat husk is mostly made up of lignocellulosic dietary fiber, which comprises the majority of its dry weight. The dietary fiber content of buckwheat husk is generally high, although it varies depending on the study and analytical methods used. Dziadek et al. [12] reported its level in buckwheat husk as 79.11 g/100 g, which is confirmed by previous research by Lu et al. [4], who detected it in the amount of 80.6 g/100 g [4,12]. Zhang et al. [5] indicated a lower level of this compound (31.31 g/100 g). These differences are primarily attributed to differences in analytical methods and fiber definitions, as Zhang et al. [5] quantified mainly insoluble non-starch polysaccharides rather than total dietary fiber [5]. Furthermore, variations in buckwheat cultivar, geographical origin, growing conditions, processing, and sample preparation may also contribute to differences in fiber composition reported across studies [4,5,12,43,44]. These studies highlight a substantial presence of hemicellulose-derived fractions such as xylan, xyloglucan, arabinoxylan, and galactoxyloglucan, in addition to pectin-type polysaccharides, which are vital for digestive health [5,22]. The soluble fiber in buckwheat husk primarily consisted of xylose (0.06 g/100 g), glucose (0.09 g/100 g), and uronic acids (0.09 g/100 g) [5].

According to Regulation (EC) No 1924/2006, products can be labelled with the nutrition claims “source of fiber” if the product contains at least 3 g of fiber per 100 g or 1.5 g of fiber per 100 kcal or “high in fiber” if it contains at least 6 g of fiber per 100 g or 3 g of fiber per 100 kcal [45]. Strategies to help meet dietary fiber consumption recommendations include adding small amounts of insoluble fiber, such as fiber-rich buckwheat husk, to foods [46], or regularly consuming whole grains, which are rich in fiber and other biologically active compounds, including vitamins, minerals, and phenolics [47]. Owing to its high insoluble fiber content, buckwheat husk may therefore represent a promising ingredient for functional foods.

The average crude protein content of buckwheat husk is approximately 4–6 g/100 g [43]. The fats constitute less than 1 g/100 g (often about 0.5 g/100 g or lower) [21]. The starch content of buckwheat husk is typically modest, varying from about 1.2 g/100 g to 2.6 g/100 g, contingent upon the source and analytical method employed [5,12]. The residual macronutrient content of the husk is relatively low. However, a recent study conducted by our team proved that adding buckwheat husk to wheat bread significantly increased the content of mineral compounds [15]. The consumption of a 100 g portion of wholemeal bread enriched with 4.5% buckwheat husk can provide up to almost 70% of the recommended daily manganese intake for adults, compared with approximately 60% for the control bread without buckwheat husk [15]. The ash content in buckwheat husk is reported to vary between around 1.5 and 2.1 g/100 g, suggesting a consistent mineral composition across several experiments [4,5,12,45]. Extractable metal ions in buckwheat husk show potassium as the main element and calcium and magnesium at modest quantities. Iron and manganese are found in lower amounts, while trace metals including zinc, copper, nickel, chromium, and silver are barely detectable. The low concentration of potentially harmful metals makes buckwheat husk suitable for food and functional ingredient uses under proper processing conditions [48].

### 3.4. Buckwheat Husk Phenolic Compounds and Other Bioactive Constituents

**Figure 1:**
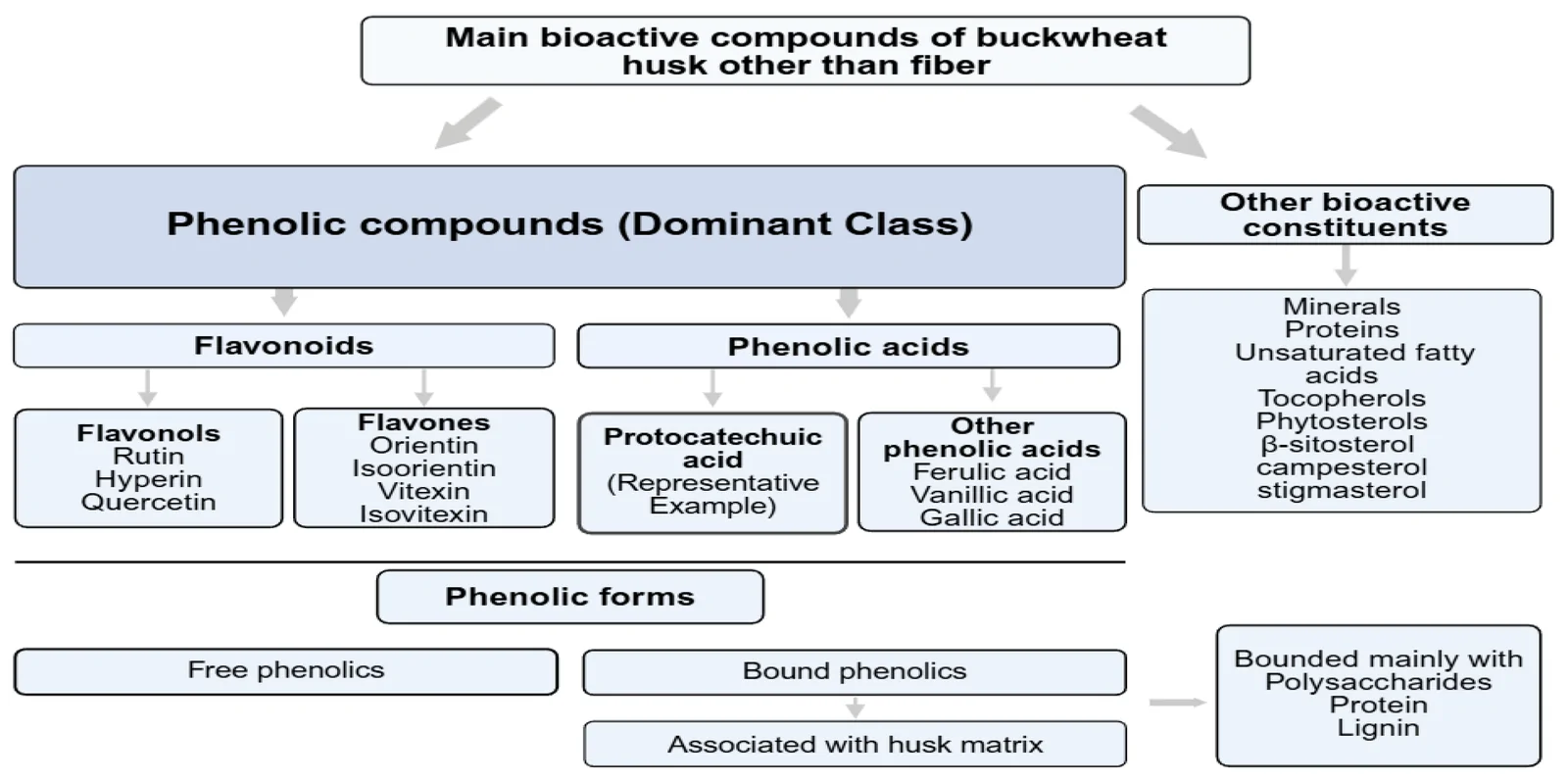
Classification of the main bioactive components of buckwheat husk other than dietary fiber.

The chemical composition of buckwheat husk comprises several groups of compounds that differ in their physicochemical characteristics and association with the husk matrix. An overview of these compounds and the distribution of phenolic compounds between free and bound forms is presented in Figure 1.

Buckwheat husk serves as a substantial source of bioactive phytochemicals [5]. The content of total phenolic compounds ranges from 434.06 to 525 mg/100 g, depending on, among others, the buckwheat variety and the conditions of its cultivation, harvesting, storage, and processing [12]. Rutin (quercetin-3-rutinoside) is generally the dominant flavonoid in common buckwheat husk. Among *Fagopyrum esculentum* cultivars, its concentration ranges from 62.43 to 173.57 mg/100 g and depends strictly on genetic and environmental factors [11,34]. Notably, Tartary buckwheat (*Fagopyrum tataricum*) contains significantly higher concentrations of rutin and associated flavonoids than common buckwheat. Although Tartary buckwheat possesses high rutinosidase activity, its seeds accumulate exceptionally high levels of rutin, approximately 100-fold higher than those of common buckwheat [49,50,51]. Using an ultrasound-assisted natural deep eutectic solvent extraction system, Huang et al. [6] achieved a maximum rutin extraction yield of 9.5 mg/g from Tartary buckwheat husk, corresponding to a leaching efficiency of 95%, and this type of process also resulted in greater extraction of other phenolic compounds. These findings show the high potential of Tartary buckwheat husk as a source of rutin for green extraction technologies [6]. Zhang et al. [11], in addition to rutin, found six other flavonoids in buckwheat husk: orientin, isoorientin, vitexin, isovitexin, hyperin, and quercetin [11]. Vitexin and isovitexin concentrations ranged from 101.65 to 188.78 mg/100 g, hyperin from 53.55 to 274.10 mg/100 g, and orientin, isoorientin, and quercetin were detected at lower concentrations [11]. Another group of phenolic compounds found in buckwheat husk is phenolic acids, such as ferulic, vanillic, protocatechuic, and gallic acid [21,52,53]. Protocatechuic acid is reported to be the dominant phenolic acid in buckwheat husk. Zhang et al. [5] measured it at the level of approximately 39 mg/100 g in raw husk, including 18.48 mg/100 g in the free form and 20.59 mg/100 g in the bound form [5]. However, earlier studies by Li et al. [54] and Zhu et al. [40] reported a higher protocatechuic acid content in dry husk (54 mg/100 g), whereas only approximately 18 mg/100 g was detected in fine bran, indicating that both the milling fraction and genotype may influence protocatechuic acid concentration [40,54]. In addition to phenolic compounds, buckwheat husk contains lipophilic bioactive constituents, including tocopherols and phytosterols. Tocopherols have been detected in buckwheat husk [55], while α-, γ-, and δ-tocopherol have been identified in husk extracts. The phytosterol fraction includes β-sitosterol, campesterol, and stigmasterol [56]. These findings showed that the bioactive composition of buckwheat husk extends beyond its extensively studied phenolic constituents. These bioactive compounds, through their antioxidant properties, provide various health benefits, including the reduction in cholesterol levels, lowering blood pressure, alleviating inflammatory responses, and assisting in the management of diabetes and obesity [57,58,59,60,61].

Phenolic compounds in buckwheat, due to the way they are bound to the cell matrix, occur in both free and bound forms [32]. Which of the above-mentioned forms dominates is influenced by the characteristics of the buckwheat varieties, their cultivation and storage conditions, as well as the method of technological treatment. The existing literature provides limited information regarding the relationships between the different forms of phenolic compounds in buckwheat husk; therefore, further discussion will utilize buckwheat grains as a representative example.

### 3.5. Factors Affecting the Chemical Composition of Buckwheat Husk

The reported composition of buckwheat husk and the main factors affecting its variation are summarized in Table 1.

Processing conditions, particularly thermal treatment, can significantly influence the phenolic composition of buckwheat. Bhinder et al. [62] conducted infrared roasting of Tartary buckwheat grains at 130, 150, and 170 °C for 10 min and observed that total phenolic content decreased with increasing roasting temperature, while the greatest losses occurred at 170 °C. Total flavonoid content remained relatively stable at 130 and 150 °C but declined markedly at 170 °C, whereas rutin was identified as the most thermally stable polyphenolic compound. The decrease in phenolic compound levels at elevated temperatures may result from thermal degradation of these compounds [62].

However, Şensoy et al. [63] reported that this phenomenon is not always evident. The authors demonstrated that extrusion of buckwheat seeds at 170 °C did not affect the content of phenolics, but roasting at 200 °C after just 10 min resulted in a change in both polar and non-polar phenolic compounds and slightly reduced antioxidant activity [63]. Some authors reported that increasing the processing temperature of buckwheat seeds may increase the content of phenolic compounds [63,64]. Liu et al. [65] reported that roasting Black Tartary buckwheat husks under optimized conditions (75 °C for 12 min) significantly increased the contents of free and bound phenolics by 8.61 and 20.88 mg gallic acid equivalents GAE/g, respectively. The authors attributed this increase to the disruption of cell-wall structures and the release of bound phenolic compounds, which enhanced their extractability [65]. Plumier et al. [66] reported that subcritical-water flash treatment of buckwheat flour at 120 °C and 140 °C lowered total phenolic content, whereas treatment at 160 °C for 30 min increased total phenolic content by 12.7% relative to raw flour [66]. The different directions of changes in the levels of these compounds observed during heating may result, on the one hand, from thermal degradation of the free fraction of phenolic compounds, which reduces their content, and on the other hand, from hydrolysis of these components’ bonds with other matrix components (e.g., proteins or saccharides), which results in an increase in polyphenolics content due to their release [49,67]. These findings indicate that the effect of thermal processing depends on, among others, the processing method and its conditions, but also on the composition of the heated product. Therefore, the strategy for best preservation of the health-promoting properties of heated products should always be evaluated individually for each of them [49,66].

The content of phenolic compounds is influenced not only by the processing parameters, but also by their type. Chen et al. [68] compared steaming, roasting, enzymatic treatment, and fermentation, and showed that fermentation exerted the greatest positive effect on the phenolic composition of both Tartary and common buckwheat. Compared with the unprocessed samples, fermentation increased the total phenolic content by 1.24-fold in Tartary buckwheat and 0.67-fold in common buckwheat, reaching 16.2 and 11.4 mg (GAE/g DW), respectively. Similarly, the total flavonoid content increased by 1.33-fold in Tartary buckwheat and 1.0-fold in common buckwheat, reaching 36.5 and 20.7 mg RE/g DW, respectively. It should be noted that total phenolic and total flavonoid contents were determined using different analytical assays and are expressed as gallic acid equivalents (GAE) and rutin equivalents (RE), respectively; therefore, their numerical values are not directly comparable. In contrast, steaming, roasting, and enzymatic treatment significantly reduced total phenolic content and bioactivity, with enzymatic treatment causing the greatest reduction in total flavonoid content, whereas roasting also decreased total phenolic content [68]. Klepacka et al. [49] showed that dehulling significantly altered the phenolic profile of buckwheat groats. Under the influence of the dehulling process, the rutin content decreased (in the range from 31% to 55%, depending on the origin of grains). The amount of coumaric, syringic, and vanillic acid increased in all grains (in the following ranges: 33–42%, 37–44%, and 16–26%, respectively) [49]. Based on these changes, the authors suggested that the largest amount of rutin occurred in the husk. The phenolic acids, coumaric, syringic, and vanillic, were predominantly located in the aleurone layer and the germ. Depending on how the dehulling process is carried out, these compounds may pass into the husk or bran or remain in the flour fraction.

Phenolic compounds distribution is not uniform throughout the grain, with the highest concentrations found in the outer tissues, particularly in the husk and bran [69]. During buckwheat processing, the husk, which constitutes approximately 17–20% of the grain, is removed during dehulling before milling. Consequently, the resulting bran and middling fractions retain substantially higher concentrations of phenolic compounds than the light flour fraction. Martín-García et al. [10] showed that the total content of free phenolic compounds in different flours obtained from buckwheat seeds can be arranged in descending order as follows: bran flour (124.25 mg/100 g DM) > medium flour (90.11 mg/100 g DM) > flour from dehulled grain (52.07 mg/100 g DM) > light flour (4.64 mg/100 g DM). The authors attributed this distribution to the higher concentration of flavonoids in the outer layers of the grain [10].

Differences in the phenolic content reported among studies can also be attributed to the buckwheat species and cultivar. Fabjan et al. (2003) reported that Tartary buckwheat seeds contained more rutin (about 0.8–1.7% DM) than common buckwheat seeds (0.01% DM) [50]. In addition to differences between buckwheat species, considerable variation has also been reported among cultivars within the same species. Kitabayashi et al. [70] evaluated 27 common buckwheat cultivars and strains and reported a wide variation in seed rutin content. The authors found that Japanese tetraploid cultivars contained higher rutin levels than diploid cultivars. Similar genotypic differences were also observed among Tartary buckwheat strains, with seed rutin content ranging from 1110 to 1950 mg/100 g DM dry weight [70].

Environmental conditions also contribute to the variability in the phenolic composition of buckwheat. The accumulation of flavonoids is influenced by environmental factors, particularly light intensity and quality [71]. Conditions of day length and solar radiation expanded the accumulation of rutin in common buckwheat (*F. esculentum*) [72,73]. Also, late buckwheat cultivars with a late flowering period had higher content of rutin than cultivars of an early flowering period [74]. Kitabayashi et al. [75] also specified that varied differences and levels of rutin content in common buckwheat have been connected to the geographic origin of the seed and also to the environmental conditions of seed growth [75]. More recently, another study estimated that using light-emitting diodes increased the content of phenolics (orientin, homoorientin, vitexin, isovitexin, and rutin) of sprouts of common buckwheat (*F. esculentum*) [76].

Besides environmental conditions, the developmental stage of the plant and the anatomical part also influence the accumulation of phenolic compounds. The inflorescences contained the highest content of total phenolics compared to the leaves, stems, or seeds of common buckwheat experimental cultivars from the Slovak collection [77]. Similarly, the highest content of total phenolics was estimated for inflorescences of common buckwheat of the Ukrainian cultivar in the phase of the beginning of flowering. Furthermore, the total phenolic content in the leaves and stems changed according to the vegetation period and plant organ, indicating that both the developmental stage and the harvested plant tissue influence phenolic accumulation in various parts of buckwheat [78].

Overall, it is possible to summarize the chemical makeup of buckwheat husk as follows: a fiber-rich matrix (>70% dietary fiber), some protein and fat, low ash content, mineral enrichment, and a variety of bioactive phytochemicals. Nutritionally, the most important seems to be the wider inclusion of buckwheat husk in food products due to its high fiber content and significant amount of antioxidant phenolic compounds, among which flavonoids (e.g., rutin) and phenolic acids predominate.

## 4. Extraction and Analytical Approaches

The recovery of bioactive components, especially phenolic compounds, from buckwheat husk is greatly dependent on the extraction method used. The course of the most important techniques is described below.

### 4.1. Conventional Extraction Methods

Polyphenols are commonly extracted utilizing such methods as solvent extraction (including maceration and percolation) or digestion [79]. All of these conventional methods have several drawbacks, including extended extraction durations, high energy use, polyphenolic degradation, and excessive solvent use. These disadvantages have prompted research into developing more efficient extraction strategies that use less solvent and energy while preserving polyphenol bioactivities [79]. Conventional extraction methods typically require large volumes of extraction solvents and rely on manual procedures that are dependent and labor-intensive. Consequently, these techniques often lack consistency [80]. Conventional methods for extracting phenolic compounds include maceration, decoction, percolation, infusion, digestion, serial exhaustive extraction, and Soxhlet extraction [81,82,83]. Maceration extraction involves soaking a pulverized sample in an appropriate solvent in a closed system, with either constant or intermittent agitation at room temperature. Although this technique is simple, it requires significant time and large volumes of solvent [84,85]. The decoction technique involves either briefly boiling plant samples or pouring boiling water over them, followed by steeping for a specified duration. The primary limitations of decoction are the presence of significant impurities in the crude extract and its inability to extract heat-sensitive or volatile compounds [86]. The percolation method is similar to maceration, as both involve placing a pulverized sample in a closed system and allowing the solvent to percolate gradually from top to bottom. Challenges include polyphenol solubility issues, limited sample size, and prolonged extraction duration [83,85,87]. Infusion is used to extract volatile plant samples that readily dissolve their phytochemicals in an organic solvent. Infusion methods exhibit limited extraction efficiency and a narrow phytochemical spectrum, primarily due to the selective recovery of water-soluble compounds. Additionally, thermal exposure can degrade thermolabile constituents [88]. Digestion represents a modified maceration technique that employs gentle heating and is primarily utilized for plant materials containing polyphenolic compounds or substances with low solubility. However, its main limitation is the risk of degradation of thermolabile compounds if the extraction temperature is not carefully controlled [88]. Serial exhaustive extraction involves fractionating crude extracts with solvents of increasing polarity, from hexane (non-polar) to butanol (polar), to obtain a broader range of phytochemicals. However, this method is unsuitable for extracting thermolabile compounds because it requires prolonged heating [88]. In the Soxhlet extraction method, pulverized samples are placed in cellulose thimbles and positioned within the extraction chamber above the collecting flask, which is situated beneath a reflux condenser [81,82,89]. However, Soxhlet extraction needs precise temperature control, as excess heat may degrade thermolabile polyphenols [90].

### 4.2. Emerging Extraction Technologies

The extraction techniques applied to buckwheat husk, including the material characteristics, target compounds, solvent systems, extraction conditions, extraction yields, phenolic recovery, and main outcomes, are summarized in Table 2.

UAE uses ultrasonic vibrations to agitate a sample immersed in a biological solvent, based on the phenomena of cavitation. During this process, bubbles caused by ultrasonic amplitude develop over time until they approach a critical threshold, resulting in a localized increase in temperature and pressure within the solids. This strong energy destroys cell membranes, which increases the efficiency of removing bioactive substances [92]. Among the eco-friendly extraction techniques, MAE produced the largest yield of polyphenols. A recent study demonstrated that MAE was implemented in two phases: following an initial extraction phase (MAE1), the solid residue underwent a further extraction phase (MAE2). In the first phase (MAE1), ground buckwheat husk was extracted using acidified water under microwave heating conditions to recover readily available phenolic compounds. In the second phase (MAE2), the remaining solid residue was re-extracted to recover phenolics still retained within the lignocellulosic matrix. The study showed that MAE2 produced the highest phenolic yield, with a 43.6% increase in polyphenols compared with the conventional extraction method, indicating the effectiveness of MAE in enhancing polyphenol recovery [79,92]. The effectiveness of MAE may be attributed to microwaves inducing dipole rotation in lignocellulosic molecules, resulting in structural breakdown and enhancing solvent penetration and solubilization of the target molecules [93,94]. Numerous studies have proven the efficacy of UAE and MAE in extracting beneficial components from diverse food by-products, including pomegranate peels, olive pomace, and grape pomace [95,96,97]. These techniques have substantial benefits, such as reduced organic solvent usage, brief extraction durations, elevated selectivity, and enhanced extraction yields, rendering them sustainable and efficient methods for valorizing agricultural waste [98]. Considering these factors, both UAE and MAE significantly reduce extraction time and solvent consumption, making them sustainable alternatives to conventional solvent-based extraction methods [99]. Their full implementation in laboratory practice may enable the determination of higher content of bioactive components in buckwheat husk than the literature data indicate, in particular the difficult-to-extract phenolic compounds.

New methods such as high-pressure processing (HPP) and pulsed electric fields (PEF) have also demonstrated enhanced phenolic recovery from buckwheat husk [30]. A direct comparison of UAE, MAE, PEF, and HPP applied to buckwheat husk showed substantial differences in phenolic recovery. HPP at 200 MPa for 8 min resulted in a TPC of 21.76 mg GAE/100 mg DW extract, compared with 16.14 mg GAE/100 mg DW extract for UAE (30 min, 4 °C), 12.63 mg GAE/100 mg DW extract for MAE (high power, 10 s), and 9.94 mg GAE/100 mg DW extract for PEF (12 kV, 10 kJ, 200 Hz and 9 μs). Among these four techniques, HPP produced the highest TPC under the conditions investigated [30]. PEF is regarded as an innovative and environmentally friendly method for extracting polyphenols from plant matrices, involving the application of a sequence of electric pulses with an enhanced electric field for brief periods of 1 to 8 s at low temperatures (22 °C to 25 °C) [100]. PEF has been employed for the extraction of phytochemicals from several plant matrices, including grape pomace, orange peel, apple pomace, pomegranate peel, and tea twigs [101,102,103,104,105]. The extraction efficiency is contingent upon various parameters, including electrode voltage, pulse width, frequency, and energy input. HPP is a nonthermal method for enzyme inactivation, microbial decontamination, and bioactive extraction. It has been universally employed for the extraction of polyphenols from plant matrices. It modifies the cellular architecture, resulting in damage or distortion of the cell membrane, which facilitates the diffusion of secondary metabolites through the principle of mass transfer, despite bioactive chemicals being low-molecular-weight entities such as polyphenols, vitamins, and pigments [106]. Qiao Lin et al. [91] conducted a study indicating that Tartary buckwheat dietary fiber (TBDF) was obtained from Tartary buckwheat husk using enzymatic hydrolysis, with the extraction process refined using the response surface technique [91]. HPP has been used to extract phenolic compounds and anthocyanins from various plant matrices [107,108,109]. However, research on the application of novel extraction technologies to buckwheat husk remains limited [30].

### 4.3. Analytical Characterization of Extracts

After extraction, analytical characterization of buckwheat husk phytochemicals is typically done by chromatographic and spectrometric methods. Analytical methods for phenolic compounds must distinguish between their free and bound forms. Researchers extract free phenolics from buckwheat matrices using aqueous organic solvents before subjecting the remaining residue to hydrolysis to release bound phenolics [10]. Bound polyphenols in Tartary buckwheat husk have been recovered through acid or alkaline hydrolysis following the removal of the free phenolic fraction. Acid hydrolysis was conducted in 2 M HCl at 80 °C for 1 h, while alkaline hydrolysis required treatment with 2 M NaOH to release bound phenolics, which were subsequently neutralized and purified. Chromatographic characterization of the recovered phenolic compounds followed. Acid hydrolysis yielded a higher recovery of bound phenolics than alkaline hydrolysis under the conditions studied [110].

Kuznetsova et al. [111] reported the determination of flavonoids (expressed as rutin) and vitamins in buckwheat husk using HPLC (high-performance liquid chromatography) with a Milichrom-5 system [111]. The authors also demonstrated correlations between phenolic compounds (especially rutin) and the antioxidant activity of buckwheat husk. This finding is supported by studies demonstrating that feeding rats with a diet supplemented with buckwheat husk leads to enhanced free radical inactivation compared to traditional feed [111]. Compared with conventional liquid chromatography, LC–MS (liquid chromatography-mass spectrometry) provides greater sensitivity, selectivity, and accuracy. It enables the identification and quantification of phenolic compounds present at very low concentrations. In addition, the mass detector facilitates the structural characterization of individual compounds in complex plant matrices [112]. A previous study revealed that phenolic profiling was done by LC-MS. The extracts contained forty-one phenolic chemicals [30]. These analytical methods ensure that extraction methods are appropriately tested for the yield, content, and bioactivity of substances derived from buckwheat husk [30].

## 5. Bioactive Properties of Buckwheat Husk Compounds

The potential health-related effects of buckwheat husk and its extracts are summarized in Table 3. Available evidence is mainly based on in vitro and animal studies, while studies on other buckwheat fractions are considered only as supporting evidence and are discussed in detail in the following subsections.

Buckwheat husk exhibits a range of biological activities, largely associated with its phenolic compounds and other bioactive constituents. These reported biological properties are discussed below according to the main types of activity.

### 5.1. Antioxidant Activity

Buckwheat husk contains a variety of bioactive substances, including phenolic compounds such as quercetin, rutin, and protocatechuic acid, which are recognized for their potent antioxidant effects. Dziadek et al. [12] found that the phenolic content of husks ranges from 434.06 to 525 mg/100 g, depending on the cultivar and strain [12]. Extracts from buckwheat husk exhibit significant radical-scavenging ability, ascribed to these chemicals. Rutin, the primary flavonol in the husk, exhibited exceptional overall antioxidant capacity in comparison studies [21,55,67]. Phenolic antioxidants present in buckwheat husk have shown the ability to reduce the generation of reactive oxygen species (ROS), malondialdehyde (MDA), and to enhance catalase (CAT) activity in cellular experiments [67].

### 5.2. Anti-Inflammatory and Metabolic Effects

Beyond antioxidant effects, buckwheat husk compounds exert notable anti-inflammatory effects. Extracts derived using “green” methods exhibited robust antioxidant activity and demonstrated notable modification of inflammatory markers in an in vitro Caco-2 cell line. These findings emphasize the prospective applications of “green” buckwheat husk extracts in the culinary and pharmaceutical sectors [79]. As shown in another study, Tartary buckwheat dietary fiber addition in the diet of obese mice significantly reduced weight gain, decreased liver index and fat-to-body ratio, alleviated hepatic steatosis, and prevented excessive fat accumulation [107]. Phenolic compounds, particularly rutin, quercetin, protocatechuic acid, orientin, isoorientin, vitexin, isovitexin, and hyperin, together with dietary fiber, contribute to numerous health benefits through their antioxidant activity, offered numerous health benefits such as lowering cholesterol levels [21,56], lowering blood pressure, and diminishing inflammatory responses [57,58,59]. These bioactive substances also help in the management of diabetes and obesity [60,61].

### 5.3. Antimicrobial and Antifungal Activity

Buckwheat husk extracts also display antimicrobial properties against a range of pathogens. According to the study by Cabarkapa et al. [117], buckwheat husk extracts with a concentration of 50 and 100 mg/mL were very good at inactivating both Gram-positive and Gram-negative bacteria. This was determined by the disk diffusion method, which evaluates antimicrobial activity by measuring the inhibition zone around treated discs. Buckwheat husk extracts at a concentration of 100 mg/mL exhibited stronger antimicrobial activity on Gram-positive bacteria like *S. aureus*, *Bacillus cereus*, and *Enterococcus faecalis* than on Gram-negative bacteria like *Salmonella choleraesuis*, *Proteus mirabilis*, and *Escherichia coli.* Antifungal activity has also been noted [117]. A previous study has also found that ethanolic extracts from buckwheat husk can stop the growth of *Aspergillus flavus* and lower the production of aflatoxin [56].

Despite the reported bioactive properties of buckwheat husk, evidence regarding the bioavailability of its bioactive compounds remains limited. Available studies have primarily assessed their bioaccessibility using in vitro gastrointestinal digestion models, while direct evidence of human bioavailability is lacking [5]. Furthermore, some biological effects reported for buckwheat husk have been shown using extracts and doses tested in vitro or in animal models. Therefore, it remains unclear whether these extract types and doses can be used directly at effective levels in functional food applications. The limited evidence on human bioavailability and practically applicable doses represents an important gap in the current literature.

## 6. Technological Functionalities of Buckwheat Husk in Food Systems

Buckwheat husk possesses several techno-functional attributes that can be utilized in the development of food products. As a substance rich in insoluble fiber, it significantly influences water management, texture, and stability in food matrices. Table 4 delineates essential functional attributes of buckwheat husk pertinent to food systems, as derived from recent research.

### 6.1. Water Holding Capacity

In a study, Wang et al. [118] discovered that buckwheat husk powder with very small particle size (10–50 µm; disrupted individual cells) exhibited higher water-binding capacity than coarser powder (100–500 µm; intact hull fragments), thereby restricting water loss and slowing bread staling [118]. Sciarini et al. [119] observed that the increased dough viscosity was associated with the high water-holding capacity of dietary fibers, including fibers derived from buckwheat husk [119]. Zhu et al. [120] demonstrated that smaller buckwheat husk particles had greater water-holding capacity [120].

### 6.2. Oil-Holding Properties

The oil-holding capacity of dietary fiber obtained from buckwheat shells surpasses that of coarse buckwheat shell powder following micropulverization. This can help stabilize lipid-containing foods [121].

### 6.3. Bulk Density

Superfine grinding of Tartary buckwheat bran powder markedly enhanced the bulk density to 0.34 g/mL and the tap density to 0.53 g/mL, yielding favorable processing attributes [121].

### 6.4. Thermal Stability and Response to Heat Processing

The effect of heating is most often analyzed in relation to phenolic compounds, and the direction and degree of the observed changes are ambiguous and depend mainly on factors such as temperature and duration, the method of heating, and the type of the heated matrix [122]. This is well illustrated, for example, by the research of Şensoy et al. [63] who reported that thermal processing did not significantly affect the total phenolic content of white or dark buckwheat flour. However, roasting dark buckwheat flour at 200 °C for 10 min slightly decreased its antioxidant activity, whereas extrusion at 170 °C did not significantly affect antioxidant activity [63].

### 6.5. Emulsifying Properties

Tartary buckwheat bran (a husk-rich fraction) exhibits high emulsifying capacity as a particle stabilizer, due to its protein, lipid, and polyphenol (rutin) content, which imparts desirable hydrophobicity and effective interfacial tension reduction [123].

### 6.6. Effects on Dough Rheology and Structure

Zhu et al. [120] showed that the finer the buckwheat husk particle, the more water-holding capacity it had [120]. Wang et al. [118] reported that the incorporation of cell-scale buckwheat husk increased water absorption in dough. Consequently, the dough exhibited higher viscoelastic moduli and greater consistency, showing improved rheological properties [118]. Another author, Ángel L. Gutiérrez et al. [124], proved that due to a higher amount of fiber in buckwheat husk, it increased dough consistency in gluten-free bread [124].

### 6.7. Effects on Food Shelf Life and Storage Stability

The extract obtained from buckwheat husk in frozen-stored meat products or mayonnaise, in both cases, helped prolong the shelf life of products [20,21].

### 6.8. Technological Effects in Meat Products

Aqueous and ethanolic extracts of buckwheat husk decreased lipid oxidation (peroxide and thiobarbituric acid reactive substances levels) and increased the induction time of chicken meatballs during refrigerated storage [20]. In frankfurter sausages, 3% buckwheat husk reduced storage loss and enhanced firmness while increasing mineral content (Mn, Ca, K, Mg) [125]. Adding 5% ground green buckwheat sprouts to horse meat patties improved moisture and fat retention, as well as phenolic content and antioxidant activity during storage [126]. Uzakov et al. [127] reported that supplementation with 1.0% buckwheat flour or 1.0% goji extract improved the oxidative stability and quality of horse meat products by reducing protein and lipid oxidation while preserving sensory properties and color characteristics [127].

Overall, the unique combination of high dietary fiber and other component content associated with functional properties makes buckwheat husk a promising functional ingredient to improve the nutritional profile and stability of foods, provided sensory and processing factors are well managed. It is also important to note that consumers especially appreciate food products whose quality and storage stability are enhanced by adding natural ingredients rather than synthetic food additives.

## 7. Current and Emerging Applications of Buckwheat Husk in Functional Foods

Consuming functional foods can be an effective strategy for the prevention and management of non-communicable diseases, as they have the potential to enhance health. Their diversity and easy availability can significantly reduce the risk of many illnesses [94]. These are foods that, in addition to their basic nutritional value, include bioactive chemicals that can modulate physiological activities and help to prevent chronic diseases [97]. With the growing popularity of functional foods, consumers are becoming increasingly aware of food quality and the health benefits associated with dietary choices [8]. Consequently, interest in healthier foods and demand for products with added functional value have increased considerably, highlighting the need for the development of novel functional food products [5]. Buckwheat husk is progressively, although still on a small scale, being repurposed into various food products to improve nutritional and functional attributes. The application of buckwheat husk and its derivatives in different food products, together with the main parameters evaluated in the reported studies, is summarized in Table 5.

### 7.1. Application of Buckwheat Husk in Bakery and Cereal-Based Products

In bakery products, buckwheat husk has been incorporated into bread as a functional ingredient to enhance mineral content. Our previous findings showed that buckwheat husk enrichment improved the mineral composition of bread, with manganese exhibiting the most pronounced increase. Consequently, husk-enriched wholemeal bread provided substantially greater dietary coverage of manganese than the control bread, supporting the use of buckwheat husk as a nutritionally valuable bakery ingredient [15]. Research demonstrates that the inclusion of buckwheat husk (1.5–4.5%) in wheat and wholemeal breads improves antioxidant activity and phenolic content. Wholemeal breads exhibit the most consistent enhancements, with syringic acid and flavonoids (rutin, catechin, orientin) as the predominant constituents. Despite the impact of elevated husk levels on color and sensory characteristics, wholemeal loaves with 1.5% and 4.5% additions retained great consumer acceptance. The study confirmed that buckwheat husk can be a potential functional component for enhancing bread quality [16]. Another study evaluated the effect of partial replacement of semolina with 0, 1, 5, 10, 15, and 20% of ground buckwheat husk on the wheat pasta, and it turned out that the addition of buckwheat husk caused an increase in fiber content in pasta from 4.31% (control pasta) to 14.15% (pasta with 20% of buckwheat husk). Furthermore, buckwheat husk-enriched cooked pasta had higher total phenolic content and antioxidant activity [128]. In pasta, buckwheat husk has been used as a fiber- and polyphenol-rich additive to enhance nutritional quality [129].

### 7.2. Application of Buckwheat Husk in Fermented Dairy Products

Buckwheat husk addition has also shown promise in fermented dairy products like yogurt. Yogurts with husk differ in terms of physicochemical and organoleptic properties. Fortification of yogurts with buckwheat and spelt husk has resulted in lowering total acidity and reducing syneresis. Addition of spelt and buckwheat husk to yoghurts reduced the color brightness and increased the intensity of the red and yellow colors. The highest number of *S. thermophilus* was found in yogurt containing 3% buckwheat husk, and the beneficial effect of the addition of husk on *L. bulgaricus* growth was shown. The type of husk determined the flavor and odor of yogurts. Spelt husk gave the yoghurts a more intense floury and grainy flavor than buckwheat husk. However, the addition of husk may be attractive to consumers due to the content of dietary fiber. This has led to experimental “functional yogurts” that incorporate buckwheat husk for its stabilizing and nutritional properties, widening dairy innovation beyond traditional fruit or grain additives [17].

### 7.3. Application of Buckwheat Husk in Functional Beverages and Infusions

Buckwheat husk is also utilized in the production of tea. Advanced glycation end-products (AGEs) are harmful molecules formed when sugars react with proteins or lipids during high-temperature processing [130], and their accumulation is linked to chronic diseases [131]. Buckwheat husk infusions provide flavonoids such as rutin, vitexin, and isovitexin. These compounds showed strong inhibitory activity against AGE formation in an in vitro study [132]. Nevertheless, the total phenolic content extracted from buckwheat husk was ~64 times lower than that of green tea, so husk teas exhibited much weaker antioxidant and antiglycation activity than green tea [31]. Tartary buckwheat tea showed higher rutin levels and stronger anti-AGE activity than common buckwheat tea, but even so, husk infusions should be used mainly as an ingredient in mixed teas or herbal infusions rather than as a single-ingredient drink. When combined with other teas, husk flavonoids can contribute to antioxidant and antiglycation effects without greatly diluting the overall phenolic content [19,133,134].

**Table 5:** Applications of buckwheat husk and its derivatives in functional food products.

Type of Food	Husk Type and Amount	Main Properties Affected by the Addition of Husk	References
Bread	Buckwheat husk (1.5%, 3.0%, 4.5%)	Mineral composition and dietary mineral coverage	[15]
Wheat and wholemeal bread	Buckwheat husk (1.5%, 3.0%, 4.5%)	Antioxidant activity, phenolic content, color and sensory characteristics	[16]
Pasta	Ground buckwheat husk (1–20%)	Fiber content, total phenolic content and antioxidant activity	[128]
Yogurt	Buckwheat husk (up to 3%)	Physicochemical, organoleptic and microbiological properties	[17]
Tea/infusion	Buckwheat hull tea infusion (1 g/100 mL)	Flavonoids, antioxidant and antiglycation activity	[19]
Pork meatballs	Buckwheat husk extract	Antioxidant properties, lipid oxidation and shelf-life preservation	[20]
Tilapia fillets	Tartary buckwheat extract with chitosan	Shelf-life extension during refrigerated storage	[135]
Chocolate cream and honeysuckle mousse	Fine buckwheat hull powder and melanin (≈1.5 g powder and 0.037 g melanin per serving)	Sensory properties, antioxidant activity and dietary fiber	[136]

### 7.4. Application of Buckwheat Husk Derivatives in Meat and Fish Products

In the context of meat products and preservation, buckwheat husk derivatives have gained attention as natural additives. Buckwheat husk extract was used to enrich fried meatballs made from ground pork. The addition of buckwheat husk antioxidants decreased lipid oxidation in stored meatballs. The highest ability to control peroxide and 2-thiobarbituric acid reactive substances values was shown for buckwheat husk extract compared with the control meatballs without buckwheat husk extract. Buckwheat husk extract showed a higher free radical scavenging activity as well as higher Fe(II) ion chelating ability, compared with butylated hydroxytoluene. These results showed that plant extracts can be used to prolong shelf life of products by protecting them against lipid oxidation and deterioration of their nutritional quality [20]. Yang et al. [135] discovered that Tartary buckwheat extract and chitosan mixes outperformed chitosan alone in increasing the shelf life of coated tilapia (*Oreochromis niloticus*) filets during 18 days of storage at 0 °C. The shelf life of fish coated with chitosan was prolonged from 6 days to 15 days when Tartary buckwheat extract was added [135].

### 7.5. Application of Buckwheat Husk in Functional Desserts

The addition of fine buckwheat husk powder and melanin to chocolate cream and honeysuckle mousse increased their sensory appeal and antioxidant potency. Optimal inclusion (~1.5 g powder and 0.037 g melanin per serving) enhanced dietary fiber [136].

### 7.6. Critical Assessment and Research Gaps in Buckwheat Husk-Based Functional Foods

While buckwheat husk can improve the nutritional and functional properties of food products, its inclusion at higher concentrations may adversely affect product quality. Substituting 1–20% ground buckwheat husk for semolina in wheat pasta increased dietary fiber, total phenolic content, and antioxidant activity. However, husk addition markedly altered product color, and incorporation levels exceeding 10% produced objectionable odor and flavor. Studies on different food products, including pasta, wheat and wholemeal bread, chocolate cream, and honeysuckle mousse, showed that a moderate amount of buckwheat husk can be incorporated while maintaining satisfactory sensory characteristics [16,129,136]. However, the appropriate level of husk addition depends on the type of food product. In pasta, additions above 10% resulted in undesirable changes in smell and taste [129]. In wheat and wholemeal breads, buckwheat husk was successfully incorporated at levels of 1.5–4.5%, with wholemeal breads containing 1.5% and 4.5% husk showing the highest overall acceptability [16]. Therefore, the principal gap is not the complete absence of sensory or visual assessment, but the lack of consistent evaluation of these parameters across different buckwheat husk food applications. These findings showed that husk incorporation level requires careful consideration in functional food development because nutritional and functional enhancements must be balanced against potential changes in sensory and visual quality. Although sensory characteristics have been addressed in some studies, the methodologies used differ significantly, limiting direct comparison of the results across food products. Consequently, future work should establish product-specific incorporation limits through standardized sensory, nutritional, and functional testing. Such an approach would help identify optimal husk levels.

## 8. Safety, Allergenicity, and Regulatory Considerations

Buckwheat husk and other grain by-products may contain mycotoxins such as aflatoxins, fumonisins, and ochratoxin A, which represent potential food safety concerns. However, the prevalence of these dangers is often limited and may be significantly decreased by appropriate farming practices, careful storage, steam treatment, and decontamination. Individuals with a documented buckwheat allergy should avoid buckwheat products; for the general population, the husk is generally harmless when properly processed [137].

### 8.1. Mycotoxins and Microbiological Safety of Buckwheat Husk

Buckwheat husk poses certain food safety challenges. Mycotoxin contamination is one of the major concerns associated with buckwheat husk. Mycotoxins are common chemical contaminants present mainly in cereal products and their by-products. As reported by Keriene et al. [138] it was shown that raw buckwheat husk (without steamed treatment) showed higher aflatoxin B1(AFB1) contents (75.8 μg/kg) than buckwheat grain, only 2.3 μg/kg AFB_1_. Overall, good agricultural and storage practices (dry, cool conditions) combined with targeted decontamination are critical to address the mycological and microbial safety of buckwheat husk-derived ingredients [138]. Mycotoxin contamination is not unique to buckwheat and can be effectively mitigated through best practices in cultivation, drying, storage, and decontamination. However, these measures may reduce the risk of fungal growth and aflatoxin biosynthesis but do not necessarily remove mycotoxins already present in contaminated husk. Therefore, direct monitoring of mycotoxin levels is necessary to confirm the safety of husk-derived food ingredients. Nobili et al. [56] observed that polyphenols extracted from buckwheat husk altered the antioxidant system of the fungal cells, although temporarily, and are mainly responsible for the inhibition of AFB_1_ production as well as of fungal growth [56]. The *in vitro* study showed that extracts rich in lipophilic compounds and polyphenols considerably inhibited the growth of Aspergillus flavus and reduced AFB_1_ production. An environmentally beneficial method of reducing fungal contamination in the field or during storage is provided by supercritical CO_2_ extraction, which produces solvent-free extracts with greater quantities of these bioactives. The study showed that buckwheat husk may both naturally regulate pollutants and absorb them [56]. Some of these fungal genera were also found by Krysińska-Traczyk et al. [139] in buckwheat grain and buckwheat grain dust, where *Penicillium* spp., *Mucor mucedo*, *Alternaria alternata*, and *Cladosporium lignicola* were the predominant genera in buckwheat grain, while *Rhodotorula rubra*, *Mucor mucedo*, *Alternaria alternata*, and *Penicillium* spp. were more abundant in buckwheat grain dust. Regarding the *Fusarium* strains, *F. culmorum* was recovered from samples of grain dust, but it was not detected in grain samples [139]. Fumonisins were reported at high levels in pseudo-cereal samples from Italy, with the highest contents (up to 567.25 μg/kg) corresponding to buckwheat samples [140]. Ochratoxin A was detected in buckwheat flour. Ochratoxin A concentrations in contaminated samples were below 0.8 µg/kg [141]. These fungi are ubiquitous in cereal environments, and their presence alone does not imply hazard unless mycotoxin levels exceed regulatory limits.

### 8.2. Allergenicity of Buckwheat and Buckwheat Husk

Buckwheat can cause IgE-mediated allergy, including severe allergic reactions and anaphylaxis. Exposure can occur when eating buckwheat food (food allergen), when producing or handling buckwheat food (occupational exposure), or when sleeping on buckwheat husk pillows (household environmental exposure) [142]. The allergy is caused by specific buckwheat allergenic proteins, mainly Fag e 1 (13S globulin), Fag e 2 (2S globulin) and Fag e 3 (7S globulin/vicilin), rather than by dust itself or gluten, since buckwheat is naturally gluten-free [138]. However, while these storage proteins are localized in the grain endosperm rather than the husk, commercial husks can still retain allergenic properties due to residual flour contamination during the mechanical dehulling process. Epidemiological studies estimate that buckwheat allergy is very rare and affects roughly 0.1–0.4% of the population in buckwheat-consuming regions, while severe reactions such as anaphylaxis occur at 0.01–0.1 cases per 100,000 people per year [143]. Although cases of buckwheat allergy are very rare, products containing buckwheat husk should be labeled for allergen awareness. Individuals with diagnosed buckwheat allergy must avoid all buckwheat-derived products, whereas for the general population buckwheat and its husk remain safe, gluten-free ingredients.

### 8.3. Antinutritional Factors in Buckwheat Husk

Although buckwheat grain, including the husk, contains a high concentration of compounds that positively impact human health, some of them may also act as antinutritional factors. Their impact on human health depends primarily on their quantity, but also on interactions with other components of the buckwheat seed. Tannins, which belong to phenolic compounds, are worth mentioning as they have a beneficial effect on health (e.g., antioxidant, anti-inflammatory, and antibacterial properties), but on the other hand, they may have an antinutritional effect by hindering the absorption of certain minerals or proteins. The negative impact on protein digestibility depends on the amount and fraction of tannins. Milling fractions with 0.1% tannins exhibit much higher digestibility than fractions with 6% tannins. Moreover, microbial fermentation and thermal processing can mitigate the inhibitory effects [144].

Phytic acid (phytate) is another antinutrient to consider. Its content in buckwheat husk depends on the method of husk removal from the buckwheat seeds; therefore, data on its content provided by different authors vary. Farooq et al. [145] determined it in buckwheat husk at 1.8%, while Kasar et al. [146] detected it at 3–3.25% and Mani et al. [46] at 18.30%. [46,145,146]. The considerable variation in phytic acid content among studies is mainly attributed to differences in the buckwheat fraction analyzed, cultivar, and geographical origin. In addition, analytical methods and processing conditions, particularly dehulling, can significantly influence phytate concentration and its distribution within the grain [147]. Although phytic acid can reduce mineral bioavailability, moderate consumption of processed husk in a diversified diet, along with appropriate treatments such as dehulling and fermentation, can mitigate these effects.

The high dietary fiber, phenolic compounds, and antioxidant activity of buckwheat husk therefore still make it a valuable ingredient for functional foods even if it also contains some compounds which can act as antinutritional ingredients. Producers of functional foods with buckwheat husk should strike a balance by leveraging its fiber and bioactives while mitigating any negative nutritional effects through careful formulation and processing. Although tannins and phytic acid can reduce digestibility at high concentrations, they also confer antioxidant, antihyperlipidemic, and anticancer benefits. As a result, many authors argue that the health-promoting effects outweigh the antinutritional concerns, particularly when buckwheat husk is incorporated in moderate amounts and processed appropriately [148].

### 8.4. Regulatory Considerations and Current Gaps

International regulations regarding buckwheat allergens differ across countries. Buckwheat is not classified as a mandatory declared allergen in either the European Union or the United States [149,150]. It remains excluded from the list of nine major food allergens. Unlike other regions, Japan mandates the declaration of buckwheat as an allergen [151]. Mycotoxin limits vary by food category. Regulation (EU) 2023/915 sets maximum permitted levels of 2 μg/kg for aflatoxin B_1_ and 4 μg/kg for total aflatoxins in the relevant cereal category within the EU. The regulation further establishes maximum thresholds of 5 μg/kg for ochratoxin A in unprocessed cereal grains and 3 μg/kg in specified cereal-derived products [152]. These regulatory limits are considerably lower than the 75.8 μg/kg AFB_1_ reported in untreated buckwheat husk. However, buckwheat husk is not specifically listed as a separate category in Regulation (EU) 2023/915. Therefore, the applicable regulatory classification should be clarified when husk is intended for use as a food ingredient [151]. Kerienė et al. (2016) reported that steaming reduced AFB_1_ contamination in buckwheat husk from 75.8 to 1.9 μg/kg [138]. However, monitoring of the final husk-derived ingredient remains necessary to confirm compliance with the applicable regulatory limit. Specific limits for buckwheat husk have not yet been established.

Buckwheat husk and husk-derived extracts may fall within the EU Novel Food framework, depending on their history of food consumption, processing method, and intended use. If significant consumption as food in the EU before 15 May 1997 cannot be demonstrated, their Novel Food status should be assessed before placing them on the EU market [153]. Where the status is uncertain, consultation with the competent national authority may be required. Regulatory status depends on multiple factors, including its history of food use, chemical composition, processing methods, and intended application. The absence of harmonized allergen-labeling requirements, standardized mycotoxin limits, and a clearly defined regulatory status for novel husk extracts hinders the development of buckwheat husk-based functional foods.

## 9. Conclusions and Prioritized Future Research Needs

Buckwheat husk is a fiber-rich by-product of buckwheat processing with considerable functional and bioactive potential. It consists predominantly of lignocellulosic dietary fiber (>70% dry matter) and contains significant levels of phenolic compounds, particularly rutin and protocatechuic acid. These compounds contribute to antioxidant, anti-inflammatory, and antimicrobial activities shown mainly in in vitro and animal models. Green extraction technologies such as ultrasound- and microwave-assisted extraction enhance phenolic recovery while reducing solvent use, supporting sustainable valorisation strategies. Technologically, buckwheat husk exhibits high water- and oil-holding capacity, thermal stability, and dough-modifying effects, enabling successful incorporation into bread, pasta, yogurt, beverages, and meat systems, where it improves fiber content, antioxidant capacity, and, in some cases, shelf life.

However, current evidence remains largely preclinical. Future research should prioritize: (i) standardization of extraction and analytical protocols; (ii) bioavailability and metabolic studies; (iii) well-designed human intervention trials; (iv) optimization of processing and inclusion levels to balance functionality and sensory quality; and (v) comprehensive safety, including mycotoxin monitoring.

Overall, buckwheat husk represents a promising ingredient for sustainable functional food development. Its valorisation aligns with circular-economy principles by transforming an underutilized agro-industrial by-product into a value-added resource for health-oriented food systems.

## Figures and Tables

**Table 1:** Reported composition of buckwheat husk and major factors affecting its variation.

Husk Component	Reported Content	Main Factors Affecting Variation	References
Dietary fiber	31.31–80.6 g/100 g	Analytical method and fiber definition; cultivar; geographical origin; growing conditions; processing and sample preparation	[4,5,12]
Protein	4–6 g/100 g	Cultivar and source of material	[4,5,12]
Fat	<1 g/100 g (often approximately 0.5 g/100 g or lower)	Source of material and analytical method	[5,12]
Starch	Approximately 1.2–2.6 g/100 g	Source of material and analytical method	[5,12]
Ash	Approximately 1.5–2.1 g/100 g	Cultivar and source of material	[4,5,12]
Total phenolic compounds	434.06–525 mg/100 g	Buckwheat variety; cultivation, harvesting, storage and processing conditions	[12]
Rutin	62.43–173.57 mg/100 g	Genetic and environmental factors	[11]
Vitexin and isovitexin	101.65–188.78 mg/100 g	Cultivar/genetic differences	[11]
Hyperin	53.55–274.10 mg/100 g	Cultivar/genetic differences	[11]
Protocatechuic acid	Approximately 39–54 mg/100 g	Genotype, milling fraction and occurrence in free and bound forms	[5,40,54]

**Table 2:** Comparative overview of extraction techniques and conditions applied to buckwheat husk.

Material and Particle Size	Targeted Compounds Extracted	Technique Used	Solvent & Solid-to-Solvent Ratio	Conditions Used	Extraction Yield and Phenolic Recovery	Main Outcomes	References
Ground buckwheat husk; particle size NR *	Phenolic compounds	Conventional solvent extraction	Acidified methanol; 0.1 g/2 mL (1:20, *w*/*v* *), extraction repeated twice	Stirring for 2 h in the dark for each extraction	TPC *: 9.68 mg GAE */g dry sample	Long extraction time and high solvent consumption; potential degradation of polyphenols	[79]
Ground buckwheat husk; particle size NR *	Phenolic compounds	Ultrasound-assisted extraction (UAE *)	Water acidified with 0.25% (*v*/*v* *) glacial acetic acid; 0.1 g/4 mL (1:40, *w*/*v* *)	15 × 1 min sonication cycles, with 1 min intervals between cycles; 16 Hz *	TPC *: 1.11 mg GAE */g dry sample	No significant enhancement of polyphenol extraction compared with acidified-water extraction under the tested conditions	[79]
Ground buckwheat husk; particle size NR *	Phenolic compounds	Microwave-assisted extraction (MAE *)	Water acidified with 0.25% (*v*/*v* *) glacial acetic acid; 8 g/320 mL (1:40, *w*/*v* *)	MAE1 *: 20 min (10 min ramp + 10 min hold), max 1500 W; MAE2 *: 17 min (3 min ramp + 14 min hold), max 1800 W; temp. limit 240 °C (IR 210 °C)	MAE1 *: TPC * 7.15 mg GAE */g; MAE2 *: TPC * 13.90 ± 0.09 mg GAE */g 43.6% higher polyphenol yield than conventional acidified-methanol extraction	Enhanced recovery of phenolic compounds retained within the lignocellulosic matrix	[79]
Common buckwheat hull (*Fagopyrum esculentum*); particle size NR *	Phenolic compounds	Pulsed electric field (PEF *)	Distilled water; hull initially rehydrated at 1:20 (*w*/*v* *)	12 kV *, 10 kJ *, 200 Hz *, 9 μs	TPC *: 9.94 mg GAE */100 mg DW extract	Enhanced phenolic recovery from buckwheat husk	[30]
Common buckwheat hull (*Fagopyrum esculentum*); particle size NR *	Phenolic compounds	High-pressure processing (HPP *)	Distilled water, hull initially rehydrated at 1:20 (*w*/*v* *)	200 MPa *, 8 min	TPC: 21.76 mg GAE */100 mg DW extract (200 MPa *, 8 min); highest HPP * extraction yield: 15.10 g/100 g (400 MPa *, 4 min)	Enhanced phenolic recovery from buckwheat husk	[30]
Tartary buckwheat husk, 80-mesh	Dietary fiber	Enzymatic hydrolysis	Sequential enzymatic treatment, solid-to-solvent ratio NR *	Husk dried at 105 °C for 4 h after grinding, optimized enzymatic conditions: pH 4.5, 16% mixed enzyme addition, 10.5 h hydrolysis	Actual TBDF * extraction purity, 84.17%	Effective removal of starch, proteins, hemicellulose and lignin, producing a purified dietary-fiber fraction. Tartary buckwheat dietary fiber obtained from Tartary buckwheat husk	[91]

*: NR, not reported; TPC, total phenolic content; GAE, gallic acid equivalents; DW, dry weight; UAE, ultrasound-assisted extraction; MAE, microwave-assisted extraction; MAE1, first microwave-assisted extraction step; MAE2, second microwave-assisted extraction step; PEF, pulsed electric field; HPP, high-pressure processing; TBDF, Tartary buckwheat dietary fiber; MPa, megapascal; kV, kilovolt; kJ, kilojoule; Hz, hertz; μs, microsecond; *w*/*v*, weight/volume; *v*/*v*, volume/volume. To overcome the limitations of conventional methods, green extraction technologies such as ultrasound-assisted extraction (UAE) and microwave-assisted extraction (MAE) have been developed [91].

**Table 3:** Health-related impacts of buckwheat husk and its extracts in experimental models.

Type of Action	Husk/Extract Type (Concentration/Solvent)	Experimental Model	Research Outcomes/Key Findings	References
Antioxidant & cytoprotective	High flavonoid fractions of common buckwheat hulls extracted with 80% ethanol (EBHF * and HBHF *; 100–200 µg mL^−1^ in vitro)	In vitro—HepG2 cell model under H_2_O_2_-induced oxidative stress	- Extracts enhanced antioxidant capacity, increased cell viability (~20–40%), reduced reactive oxygen species and malondialdehyde levels, and increased catalase activity.	[67]
Hepatoprotective	Flavonoid-rich extract of common buckwheat husk (70% ethanol) which contains ≈ 4–6% crude protein and 5–10% total flavonoids	Animal study- Type 2 diabetic rats	- Reduced liver fat and lower levels of blood alanine aminotransferase and aspartate aminotransferase, which are indicators of liver health. These findings emphasize the powerful antioxidant and hepatoprotective properties of buckwheat hull flavonoids, showing that they might be useful nutraceuticals and dietary supplements for preventing and/or treating liver diseases.	[113]
Supporting weight loss and beneficial for diabetes	Flavonoid-rich buckwheat husk extract obtained by hot water extraction (121 °C, 20 min), then purification using D-101 and ADS-7 macroporous resins. And elution with 70% and 40% ethanol.	Animal study- Type 2 diabetic rats	- Mitigated insulin resistance. - Reduced blood glucose levels and improved oxidative stress responses.	[113]
Promotion of satiety and digestive health	Buckwheat husk rich in dietary fiber and phenolic compounds (insoluble fiber ~79 g per 100 g; high in xylose, glucose, and uronic acid fractions).	Human studies of buckwheat foods—indirect evidence. Nutritional studies and human trials with fiber-rich buckwheat foods	- Buckwheat husk is a particularly rich source of insoluble dietary fiber (~79% of dry weight) which, among other things, improves the functioning of the digestive tract and has a wide spectrum of health-promoting effects proven in many studies on this fiber fraction, regardless of its origin. - The high dietary fiber content of buckwheat husk supports its potential use as a fiber-rich food ingredient [16]. Human studies investigating satiety have used buckwheat foods rather than isolated husk [108,109]. Therefore, these studies provide only indirect evidence regarding the potential satiety effects of buckwheat husk. - Research shows that soluble fiber-rich foods had lower glycemic indices (GI) than high-GI foods, which is associated with fewer insulin spikes and longer periods of satiety.	[5,12]
Lowering blood glucose levels and controlling hunger	Buckwheat flour (50% incorporation into wheat bread); the study did not specify whether whole-grain flour or husk-containing flour was used.	Randomized crossover trials in 20 healthy adults.	- Significant reduction in blood glucose levels at 30 min (*p* = 0.001) and 45 min (*p* = 0.003) after consuming buckwheat bread as compared to control wheat bread without buckwheat addition. - Reduced hunger levels after buckwheat bread consumption (*p* = 0.035 and *p* = 0.0347). - High enjoyment of buckwheat bread with no gastrointestinal issues reported. - Buckwheat-containing bread decreased appetite and enhances postprandial glycemia compared to control wheat bread.	[114]
Antifungal and anti-aflatoxin	Polyphenol extract (PE, 500 μg mL^−1^) and lipophilic extract (LE, 10 μg mL^−1^) from buckwheat hulls obtained by supercritical CO_2_ extraction	*Aspergillus flavus* cultures producing aflatoxin B_1_	- * PE completely inhibited aflatoxin B_1_ production. - * LE and * PE/* LE combinations reduced mycelial growth by 74–86%. - It also suppressed aflatoxin biosynthesis.	[56]
Protease inhibition	Extracts from Tartary and common buckwheat hulls (methanol extraction for 24 h at room temperature, flavonoid content 0.1–0.6% of husk dry weight)	In vitro assays against thrombin, urokinase, elastase and trypsin	- Husk extracts inhibited thrombin and urokinase, mildly inhibited elastase, and had no impact on trypsin. - Inhibition related to total flavonoid concentration (Madawaska cultivar > Bamby > KASHO-2).	[115]
Anticancer	Buckwheat husk extracted with 70% ethanol and then further fractionated with n-hexane, chloroform, ethyl acetate, and water stepwise.	Study in vitro, SRB * assay	- Hexane and ethyl acetate fractions exhibited greater inhibitory effects on MCF-7 * and Hep3B * cells. - The ethyl acetate fraction demonstrated the best inhibition rate against A549 cells *. - It also reduced tumor development in sarcoma-180 implanted mice.	[116]

* ADS-7—polar macroporous adsorption resin, * EBHF, enriched buckwheat hull flavonoids, * HBHF, high buckwheat hull flavonoids, * MCF-7, human breast adenocarcinoma; * Hep3B, human hepatocellular carcinoma; * A549, human lung adenocarcinoma; * SRB, Sulforhodamine B, * LE, Lipophilic extract * PE, Polyphenol extract.

**Table 4:** Key techno-functional attributes of buckwheat husk and their technological relevance in food matrices.

Property	Product	Husk Type	Main Improvements	References
Water-holding capacity	Bread/dough	Fine buckwheat husk powder	Increased water binding, reduced water loss and staling	[118,119,120]
Oil-holding capacity	Lipid-containing foods	Micronized buckwheat hull dietary fiber	Increased oil-binding capacity	[121]
Bulk density	Tartary buckwheat bran powder	Superfine-ground Tartary buckwheat bran	Increased bulk and tap density	[121]
Thermal stability	Buckwheat flour	White and dark buckwheat flour	Phenolic content maintained; antioxidant activity slightly reduced by roasting.	[63,122]
Emulsifying capacity	Emulsion systems	Tartary buckwheat bran	Improved emulsion stabilization	[123]
Dough rheology	Gluten-free bread dough	Fine/cell-scale buckwheat husk	Increased water absorption, viscoelasticity and consistency	[118,120,124]
Shelf-life impact	Frozen meat products, mayonnaise	Buckwheat husk extracts	Prolonged shelf life	[20,21]
Quality and durability	Chicken meatballs	Aqueous and ethanolic husk extracts	Reduced lipid oxidation, increased induction time	[20]
Quality and durability	Frankfurter sausages	3% buckwheat husk	Reduced storage loss, increased firmness and mineral content	[125]
Quality and durability	Horse-meat patties	5% ground green buckwheat sprouts	Improved moisture/fat retention and antioxidant properties	[126]
Quality and durability	Horse-meat products	1.0% buckwheat flour or 1.0% goji extract	Reduced oxidation, maintained sensory and color properties	[127]

## Data Availability

No new data were created or analyzed in this study. Data sharing is not applicable to this article.

## References

[1] Rosentrater K.A., Evers A.D. (2018). Introduction to cereals and pseudocereals and their production. Kent’s Technology of Cereals: An Introduction for Students of Food Science and Agriculture.

[2] Sinkovič L., Kokalj Sinkovič D., Meglič V. (2021). Milling fractions composition of common (*Fagopyrum esculentum* Moench) and Tartary (*Fagopyrum tataricum* (L.) Gaertn.) buckwheat. Food Chem..

[3] FAOSTAT FAOSTAT 2016–2018. http://www.fao.org/food-agriculture-statistics/en/.

[4] Lu L., Murphy K., Baik B.K. (2013). Genotypic Variation in Nutritional Composition of Buckwheat Groats and Husks. Cereal Chem..

[5] Zhang Z., Fan S., Duncan G.J., Morris A., Henderson D., Morrice P., Russell W.R., Duncan S.H., Neacsu M. (2023). Buckwheat (*Fagopyrum esculentum*) hulls are a rich source of fermentable dietary fibre and bioactive phytochemicals. Int. J. Mol. Sci..

[6] Huang Y., Feng F., Jiang J., Qiao Y., Wu T., Voglmeir J., Chen Z.-G. (2017). Green and efficient extraction of rutin from Tartary buckwheat hull by using natural deep eutectic solvents. Food Chem..

[7] Huda M.N., Lu S., Jahan T., Ding M., Jha R., Zhang K., Zhang W., Georgiev M.I., Park S.U., Zhou M. (2021). Treasure from garden: Bioactive compounds of buckwheat. Food Chem..

[8] Krkošková B., Mrazová Z. (2005). Prophylactic components of buckwheat. Food Res. Int..

[9] Steadman K.J., Burgoon M.S., Lewis B.A., Edwardson S.E., Obendorf R.L. (2001). Buckwheat Seed Milling Fractions: Description, Macronutrient Composition and Dietary Fibre. J. Cereal Sci..

[10] Martín-García B., Pasini F., Verardo V., Gómez-Caravaca A.M., Marconi E., Caboni M.F. (2019). Distribution of free and bound phenolic compounds in buckwheat milling fractions. Foods.

[11] Zhang W., Zhu Y., Liu Q., Bao J., Liu Q. (2017). Identification and quantification of polyphenols in hull, bran and endosperm of common buckwheat (*Fagopyrum esculentum*) seeds. J. Funct. Foods.

[12] Dziadek K., Kopeć A., Pastucha E., Piątkowska E., Leszczyńska T., Pisulewska E., Witkowicz R., Francik R. (2016). Basic chemical composition and bioactive compounds content in selected cultivars of buckwheat whole seeds, dehulled seeds and hulls. J. Cereal Sci..

[13] Lee H., Lim T., Kim J., Kim R.H., Hwang K.T. (2022). Phenolics in buckwheat hull extracts and their antioxidant activities on bulk oil and emulsions. J. Food Sci..

[14] Bączek N., Haros C.M., Wronkowska M. (2023). Buckwheat hull, a valuable bakery product ingredient: Assessment of bioaccessible phenolics and antioxidant capacity. Eur. Food Res. Technol..

[15] Mumtaz W., Klepacka J., Czarnowska-Kujawska M. (2025). Modification of mineral content in bread with the addition of buckwheat husk. Appl. Sci..

[16] Mumtaz W., Czarnowska-Kujawska M., Klepacka J. (2025). Effect of buckwheat husk addition on antioxidant activity, phenolic profile, color, and sensory characteristics of bread. Molecules.

[17] Znamirowska A., Sajnar K., Kowalczyk M., Kluz M., Buniowska M. (2020). Effect of addition of spelt and buckwheat hull on selected properties of yoghurt. J. Microbiol. Biotechnol. Food Sci..

[18] Liu D., Song S., Tao L., Yu L., Wang J. (2022). Effects of common buckwheat bran on wheat dough properties and noodle quality compared with common buckwheat hull. LWT.

[19] Zielińska D., Szawara-Nowak D., Zieliński H. (2013). Antioxidative and anti-glycation activity of buckwheat hull tea infusion. Int. J. Food Prop..

[20] Hęś M., Szwengiel A., Dziedzic K., Le Thanh-Blicharz J., Kmiecik D., Górecka D. (2017). The effect of buckwheat hull extract on lipid oxidation in frozen-stored meat products. J. Food Sci..

[21] Park B.I., Kim J., Lim T., Hwang K.H. (2019). Flavonoids in common and Tartary buckwheat hull extracts and antioxidant activity of the extracts against lipids in mayonnaise. J. Food Sci. Technol..

[22] Sofi S.A., Ahmed N., Farooq A., Rafiq S., Zargar S.M., Kamran F., Dar T.A., Mir S.A., Dar B.N., Mousavi Khaneghah A. (2023). Nutritional and bioactive characteristics of buckwheat, and its potential for developing gluten-free products: An updated overview. Food Sci. Nutr..

[23] Roberfroid M.B. (2000). Concepts and strategy of functional food science: The European perspective. Am. J. Clin. Nutr..

[24] Wijngaard H.H., Arendt E.K. (2006). Buckwheat. Cereal Chem..

[25] Kreft M. (2016). Buckwheat phenolic metabolites in health and disease. Nutr. Res. Rev..

[26] Mikami T., Motonishi S., Tsutsui S. (2018). Production, uses and cultivars of common buckwheat in Japan: An overview. Acta Agric. Slov..

[27] Kowalska E., Ziarno M. (2020). Characterization of buckwheat beverages fermented with lactic acid bacterial cultures and bifidobacteria. Foods.

[28] Liu Q., Yao H. (2007). Antioxidant activities of barley seeds extracts. Food Chem..

[29] Lee L.-S., Choi E.-J., Kim C.-H., Sung J.-M., Kim Y.-B., Seo D.-H., Choi H.-W., Choi Y.-S., Kum J.-S., Park J.-D. (2016). Contribution of flavonoids to the antioxidant properties of common and tartary buckwheat. J. Cereal Sci..

[30] Noore S., Joshi A., Kumari B., Zhao M., O’Donnell C., Tiwari B.K. (2022). Effects of Novel Extraction Strategies on the Recovery of Phenolic Compounds and Associated Antioxidant Properties from Buckwheat Hull (*Fagopyrum esculentum*). Processes.

[31] Xu Q., Wang L., Li W., Xing Y., Zhang P., Wang Q., Li H., Liu H., Yang H., Liu X. (2019). Scented Tartary buckwheat tea: Aroma components and antioxidant activity. Molecules.

[32] Gimenez-Bastida J.A., Piskula M.K., Zielinski H. (2015). Recent advances in processing and development of buckwheat-derived bakery and nonbakery products: A review. Pol. J. Food Nutr. Sci..

[33] Wronkowska M., Szawara-Nowak D., Zielińska D., Troszyńska A., Soral-Śmietana M. (2009). Influence of the addition of buck-wheat flour on gluten-free bread quality and antioxidant capacity. Czech J. Food Sci..

[34] Atambayeva Z., Nurgazezova A., Amirkhanov K., Assirzhanova Z., Khaimuldinova A., Charchoghlyan H., Kaygusuz M. (2024). Unlocking the potential of buckwheat hulls, sprouts, and extracts: Innovative food product development, bioactive compounds, and health benefits—A review. Pol. J. Food Nutr. Sci..

[35] Ahmed A., Khalid N., Ahmad A., Abbasi N.A., Latif M.S.Z., Randhawa M.A. (2014). Phytochemicals and biofunctional properties of buckwheat: A review. J. Agric. Sci..

[36] Roy M., Dutta H., Jaganmohan R., Choudhury M., Kumar N., Kumar A. (2019). Effect of steam parboiling and hot soaking treatments on milling yield, physical, physicochemical, bioactive and digestibility properties of buckwheat (*Fagopyrum esculentum* L.). J. Food Sci. Technol..

[37] Sinkovič L., Pipan B., Neji M., Rakszegi M., Meglič V. (2023). Influence of hulling, cleaning and brushing/polishing of (pseudo)cereal grains on compositional characteristics. Foods.

[38] Kwon S.J., Roy S.K., Choi J., Park J., Cho S., Sarker K., Woo S.H. (2018). Recent research updates on functional components in buckwheat. J. Agric. Sci.-Chungbuk Natl. Univ..

[39] Luthar Z., Golob A., Germ M., Vombergar B., Kreft I. (2021). Tartary buckwheat in human nutrition. Plants.

[40] Zhu F. (2016). Chemical composition and health effects of Tartary buckwheat. Food Chem..

[41] Pocienė O., Šlinkšienė R. (2022). Studies on the possibilities of processing buckwheat husks and ash in the production of environmentally friendly fertilizers. Agriculture.

[42] Kumar D., Kalita P. (2017). Reducing postharvest losses during storage of grain crops to strengthen food security in developing countries. Foods.

[43] Bonafaccia G., Marocchini M., Kreft I. (2003). Composition and technological properties of the flour and bran from common and Tartary buckwheat. Food Chem..

[44] Wiśniewska M., Mańkowski D.R., Fraś A. (2024). Variations in chemical composition of common buckwheat (*Fagopyrum esculentum* Moench) as a result of different environmental conditions. J. Sci. Food Agric..

[45] European Parliament, Council of the European Union (2006). Regulation (EC) No 1924/2006 of the European Parliament and of the Council of 20 December 2006 on nutrition and health claims made on foods. Off. J. Eur. Union.

[46] Mishra M., Jain S. (2019). A comparative study on nutritional profile and antinutrients of buckwheat fractions (*Fagopyrum esculentum*). Int. J. Curr. Microbiol. Appl. Sci..

[47] Mattila P.H., Pihlava J.M., Hellström J., Nurmi M., Eurola M., Mäkinen S., Pihlanto A. (2018). Contents of phytochemicals and anti-nutritional factors in commercial protein-rich plant products. Food Qual. Saf..

[48] Zemnukhova L.A., Shkorina E., Fedorishcheva G.A. (2005). Composition of inorganic components of buckwheat husk and straw. Russ. J. Appl. Chem..

[49] Klepacka J., Najda A. (2021). Effect of commercial processing on polyphenols and antioxidant activity of buckwheat seeds. Int. J. Food Sci. Technol..

[50] Fabjan N., Rode J., Košir I.J., Wang Z., Zhang Z., Kreft I. (2003). Tartary buckwheat (*Fagopyrum tataricum* Gaertn.) as a source of dietary rutin and quercitrin. J. Agric. Food Chem..

[51] Suzuki T., Morishita T., Kim S.-J., Park S.U., Woo S.-H., Noda T., Takigawa S. (2015). Physiological roles of rutin in the buckwheat plant. Jpn. Agric. Res. Q..

[52] Lim J.H., Park K.J., Kim B.K., Jeong J.W., Kim H.J. (2012). Effect of salinity stress on phenolic compounds and carotenoids in buckwheat (*Fagopyrum esculentum* M.) sprout. Food Chem..

[53] Lin L.Y., Peng C.C., Yang Y.L., Peng R.Y. (2008). Optimization of bioactive compounds in buckwheat sprouts and their effect on blood cholesterol in hamsters. J. Agric. Food Chem..

[54] Li H., Wang J., Sun B., Tsao R. (2019). Buckwheat. Bioactive Factors and Processing Technology for Cereal Foods.

[55] Sedej I., Sakač M., Mandić A., Mišan A., Tumbas V., Čanadanović-Brunet J. (2012). Buckwheat (*Fagopyrum esculentum* Moench) grain and fractions: Antioxidant compounds and activities. J. Food Sci..

[56] Nobili C., De Acutis A., Reverberi M., Bello C., Leone G.P., Palumbo D., Natella F., Procacci S., Zjalic S., Brunori A. (2019). Buckwheat Hull Extracts Inhibit Aspergillus flavus Growth and AFB1 Biosynthesis. Front. Microbiol..

[57] Al-Khayri J.M., Sahana G.R., Nagella P., Joseph B.V., Alessa F.M., Al-Mssallem M.Q. (2022). Flavonoids as potential anti-inflammatory molecules: A review. Molecules.

[58] Qing L., Li S., Yan S., Wu C., Yan X., He Z., Chen Q., Huang M., Shen C., Wang S. (2023). Anti-*Helicobacter pylori* activity of *Fagopyrum tataricum* (L.) Gaertn. bran flavonoid extract and its effect on *Helicobacter pylori*-induced inflammatory response. Food Sci. Nutr..

[59] Tsai H., Deng H., Tsai S., Hsu Y. (2012). Bioactivity comparison of extracts from various parts of common and Tartary buckwheats: Evaluation of the antioxidant and angiotensin-converting enzyme inhibitory activities. Chem. Cent. J..

[60] Cai C., Cheng W., Shi T., Liao Y., Zhou M., Liao Z. (2023). Rutin alleviates colon lesions and regulates gut microbiota in diabetic mice. Sci. Rep..

[61] Zhao Z.Y., Piao C.H., Wang Y.H., Liu J.M., Yu H.S., Dai W.C., Tang Y.F., Wang J., Liu D.L. (2018). Isolation and anti-diabetic activity in vitro of flavonoids from buckwheat hull. Food Sci..

[62] Bhinder S., Singh B., Kaur A., Singh N., Kaur M., Kumari S., Yadav M.P. (2019). Effect of infrared roasting on antioxidant activity, phenolic composition and Maillard reaction products of Tartary buckwheat varieties. Food Chem..

[63] Sensoy I., Rosen R.T., Ho C.-T., Karwe M.V. (2006). Effect of processing on buckwheat phenolics and antioxidant activity. Food Chem..

[64] Zhu F. (2026). Buckwheat grain phenolics: Composition, bioactivity, and processing. Food Chem..

[65] Liu A., Xing Y., Yang P., Ma Y., Xu Q., Liu H., He L., Yang L., Dong S. (2025). Effects of roasting treatment on the composition, digestive properties, and antioxidant activities of free and bound phenolics in black Tartary buckwheat husks. J. Food Sci..

[66] Plumier B., Kenar J.A., Felker F.C., Winkler-Moser J., Singh M., Byars J.A., Liu S.X. (2023). Effect of subcritical water flash release processing on buckwheat flour properties. J. Sci. Food Agric..

[67] Cui Y., Zhao Z., Liu Z., Liu J., Piao C., Liu D. (2020). Purification and identification of buckwheat hull flavonoids and its comparative evaluation on antioxidant and cytoprotective activity in vitro. Food Sci. Nutr..

[68] Chen S., Wu L., Zhu H., Yao L., Wang L. (2021). Effects of processing methods on phenolic compositions, antioxidant activities and α-glucosidase inhibitory ability of two buckwheat varieties. Chem. Pap..

[69] Saturni L., Ferretti G., Bacchetti T. (2010). The gluten-free diet: Safety and nutritional quality. Nutrients.

[70] Kitabayashi H., Ujihara A., Hirose T., Minami M. (1995). On the genotypic differences for rutin content in Tartary buckwheat, *Fagopyrum tataricum* Gaertn. Jpn. J. Breed..

[71] Tegelberg R., Julkunen-Tiitto R., Aphalo P.J. (2004). Red:far-red light ratio and UV-B radiation: Their effects on leaf phenolics and growth of silver birch seedlings. Plant Cell Environ..

[72] Ohara T., Ohinata H., Muramatsu N., Matsuhashi T. (1989). Determination of rutin in buckwheat foods by high-performance liquid chromatography. J. Food Sci. Technol.-Mysore.

[73] Ohsawa R., Tsutsumi T., Matano T., Ujihara A. (1995). Improvement of rutin content in buckwheat flour. Current Advances in Buckwheat Research.

[74] Oomah B.D., Mazza G. (1996). Flavonoids and antioxidative activities in buckwheat. J. Agric. Food Chem..

[75] Kitabayashi H., Ujihara A., Hirose T., Minami M. (1995). Varietal differences and heritability for rutin content in common buckwheat, *Fagopyrum esculentum* Moench. Breed. Sci..

[76] Hossen Z. (2007). Light emitting diodes increase phenolics of buckwheat (*Fagopyrum esculentum*) sprouts. J. Plant Interact..

[77] Bystrická J., Vollmannová A., Margitanová E., Čičová I. (2010). Dynamics of polyphenolics formation in different plant parts and different growth phases of selected buckwheat cultivars. Acta Agric. Slov..

[78] Sytar O., Borankulova A., Hemmerich I., Rauh C., Smetanska I. (2014). Effect of chlorocholine chloride on phenolic acids accumulation and polyphenols formation of buckwheat plants. Biol. Res..

[79] Speranza A.R., Ghidotti F.G., Barbiroli A., Scarafoni A., Limbo S., Iametti S. (2025). Setting up a green extraction protocol for bioactive compounds in buckwheat husk. Int. J. Mol. Sci..

[80] Alara O.R., Abdurahman N.H., Ukaegbu C.I. (2021). Extraction of phenolic compounds: A review. Curr. Res. Food Sci..

[81] Alara O.R., Abdurahman N.H., Ukaegbu C.I. (2018). Soxhlet extraction of phenolic compounds from Vernonia cinerea leaves and its antioxidant activity. J. Appl. Res. Med. Aromat. Plants.

[82] Alara O.R., Abdurahman N.H., Ukaegbu C.I., Azhari N.H. (2018). Vernonia cinerea leaves as the source of phenolic compounds, antioxidants, and anti-diabetic activity using microwave-assisted extraction technique. Ind. Crop. Prod..

[83] Kaufmann B., Christen P. (2002). Recent extraction techniques for natural products: Microwave-assisted extraction and pressurised solvent extraction. Phytochem. Anal..

[84] Olejar K.J., Fedrizzi B., Kilmartin P.A. (2015). Influence of harvesting technique and maceration process on aroma and phenolic attributes of Sauvignon blanc wine. Food Chem..

[85] Sticher O. (2008). Natural product isolation. Nat. Prod. Rep..

[86] Kumar A., P N., Kumar M., Jose A., Tomer V., Oz E., Proestos C., Zeng M., Elobeid T., K S. (2023). Major Phytochemicals: Recent Advances in Health Benefits and Extraction Method. Molecules.

[87] Ojong C., Besong S.A., Aryee A.N.A. (2026). Solvent-Based Extraction Recovers Phytochemicals from Medicinal Plants Demonstrating Anticancer and Chemopreventive Potential: A Review. Molecules.

[88] Alara O.R., Abdurahman N.H. (2019). Kinetics studies on effects of extraction techniques on bioactive compounds from Vernonia cinerea leaf. J. Food Sci. Technol..

[89] Luque de Castro M.D., García-Ayuso L.E. (1998). Soxhlet extraction of solid materials: An outdated technique with a promising innovative future. Anal. Chim. Acta.

[90] Seidel V., Sarker S.D., Nahar L. (2012). Initial and bulk extraction of natural products isolation. Natural Products Isolation.

[91] Lin Q., Ji C., Yao X., Hua X., Zhu Y., Deng Y., Chen Y., Liu R., Qiu J. (2025). Extraction, characterization, and impact of Tartary buckwheat husk dietary fiber on metabolic functions and gut microbiota composition in obese mice. Qual. Assur. Saf. Crops Foods.

[92] Pagano I., Campone L., Celano R., Piccinelli A.L., Rastrelli L. (2021). Green non-conventional techniques for the extraction of polyphenols from agricultural food by-products: A review. J. Chromatogr. A.

[93] Fernandes A., Cruz-Lopes L., Esteves B., Evtuguin D.V. (2023). Microwaves and ultrasound as emerging techniques for lignocellulosic materials. Materials.

[94] Antón M., Aranibar C., Dusso D., Moyano L., Aguirre A., Borneo R. (2023). Exploring green extraction methods to obtain polyphenols from partially defatted chia (*Salvia hispanica* L.) flour. Expl. Foods Foodomics.

[95] Singh N., Patle D.S., Kumar S. (2024). Microwave- and ultrasonication-based intensified and synergetic approaches for extraction of bioactive compounds from pomegranate peels: Parametric and kinetic studies. Ind. Eng. Chem. Res..

[96] Stramarkou M., Missirli T.-V., Kyriakopoulou K., Papadaki S., Angelis-Dimakis A., Krokida M. (2023). The recovery of bioactive compounds from olive pomace using green extraction processes. Resources.

[97] Drosou C., Kyriakopoulou K., Bimpilas A., Tsimogiannis D., Krokida M. (2015). A comparative study on different extraction techniques to recover red grape pomace polyphenols from vinification byproducts. Ind. Crops Prod..

[98] Osorio-Tobón J.F. (2020). Recent advances and comparisons of conventional and alternative extraction techniques of phenolic compounds. J. Food Sci. Technol..

[99] Vinatoru M., Calinescu I. (2017). Ultrasonically assisted extraction (UAE) and microwave-assisted extraction (MAE) of functional compounds from plant materials. TrAC Trends Anal. Chem..

[100] Donsì F., Ferrari G., Pataro G. (2010). Applications of pulsed electric field treatments for the enhancement of mass transfer from vegetable tissue. Food Eng. Rev..

[101] Balasa A., Toepfl S., Knorr D. (2006). Impact of pulsed electric field treatment on polyphenolic content of grapes. Control Applications in Post-Harvest and Processing Technology (CAPPT 2006).

[102] Luengo E., Álvarez I., Raso J. (2013). Improving the pressing extraction of polyphenols of orange peel by pulsed electric fields. Innov. Food Sci. Emerg. Technol..

[103] Wiktor A., Sledz M., Nowacka M., Rybak K., Chudoba T., Lojkowski W., Witrowa-Rajchert D. (2015). The impact of pulsed electric field treatment on selected bioactive compound content and color of plant tissue. Innov. Food Sci. Emerg. Technol..

[104] Rajha H.N., Abi-Khattar A.-M., El Kantar S., Boussetta N., Lebovka N., Maroun R.G., Louka N., Vorobiev E. (2019). Comparison of aqueous extraction efficiency and biological activities of polyphenols from pomegranate peels assisted by infrared, ultrasound, pulsed electric fields and high-voltage electrical discharges. Innov. Food Sci. Emerg. Technol..

[105] Zderic A., Zondervan E., Meuldijk J. (2013). Breakage of cellular tissue by pulsed electric field: Extraction of polyphenols from fresh tea leaves. Chem. Eng. Trans..

[106] Knorr D. (1993). Effects of high-hydrostatic-pressure processes on food safety and quality. Food Technol..

[107] Chemat F., Vian M.A., Cravotto G. (2012). Green extraction of natural products: Concept and principles. Int. J. Mol. Sci..

[108] Altuner E.M., Işlek C., Çeter T., Alpas H. (2012). High hydrostatic pressure extraction of phenolic compounds from *Maclura pomifera* fruits. Afr. J. Biotechnol..

[109] Corrales M., Toepfl S., Butz P., Knorr D., Tauscher B. (2008). Extraction of anthocyanins from grape by-products assisted by ultrasonics, high hydrostatic pressure or pulsed electric fields: A comparison. Innov. Food Sci. Emerg. Technol..

[110] Dzah C.S., Duan Y., Zhang H., Ma H. (2021). Effects of pretreatment and type of hydrolysis on the composition, antioxidant potential and HepG2 cytotoxicity of bound polyphenols from Tartary buckwheat (*Fagopyrum tataricum* L. Gaerth) hulls. Food Res. Int..

[111] Kuznetsova E., Uchasov D., Kuznetsova O., Bychkova T., Brindza J. The use of high-performance liquid chromatography (HPLC) to assess the antioxidant activity of buckwheat husk and indicators of the oxidant-antioxidant system of laboratory animals. Proceedings of the International Scientific Conference-Digital Transformation on Manufacturing, Infrastructure and Service.

[112] López-Fernández O., Domínguez R., Pateiro M., Munekata P.E.S., Rocchetti G., Lorenzo J.M. (2020). Determination of polyphenols using liquid chromatography–tandem mass spectrometry technique (LC–MS/MS): A review. Antioxidants.

[113] Wang H., Liu S., Cui Y., Wang Y., Guo Y., Wang X., Liu J., Piao C. (2021). Hepatoprotective effects of flavonoids from common buckwheat hulls in type 2 diabetic rats and HepG2 cells. Food Sci. Nutr..

[114] Begum K., Khan I., Al-Rizeiqi M.H., Johnson S.K., Almajwal A.M. (2025). Effect of buckwheat-containing bread on postprandial glycemia, appetite, palatability, and gastrointestinal well-being. Food Sci. Nutr..

[115] Danihelová M., Šturdík E. (2013). Antioxidant and antiproteinase effects of buckwheat hull extracts. Potravinarstvo.

[116] Kim S.-H., Cui C.-B., Kang I.-J., Kim S.Y., Ham S.-S. (2007). Cytotoxic effect of buckwheat (*Fagopyrum esculentum* Moench) hull against cancer cells. J. Med. Food.

[117] Čabarkapa I.S., Sedej I.J., Sakač M.B., Šarić L.Č., Plavšić D. (2008). Antimicrobial activity of buckwheat (*Fagopyrum esculentum* Moench) hull extract. Food Process. Qual. Saf..

[118] Wang L., Li Y., Guo Z., Wang H., Wang A., Li Z., Chen Y., Qiu J. (2022). Effect of buckwheat hull particle size on bread staling quality. Food Chem..

[119] Sciarini L., Ribotta P., León A. (2010). Influence of gluten-free flours and their mixtures on batter properties and bread quality. Food Bioprocess Technol..

[120] Zhu F., Du B., Li R., Li J. (2014). Effect of micronization technology on physicochemical and antioxidant properties of dietary fiber from buckwheat hulls. Biocatal. Agric. Biotechnol..

[121] Xu Q., Huang R., Yang P., Wang L., Xing Y., Liu H., Wu L., Che Z., Zhang P. (2021). Effect of different superfine grinding technologies on the physicochemical and antioxidant properties of Tartary buckwheat bran powder. RSC Adv..

[122] Liu Y., Cai C., Yao Y., Xu B. (2019). Alteration of phenolic profiles and antioxidant capacities of common buckwheat and Tartary buckwheat produced in China upon thermal processing. J. Sci. Food Agric..

[123] Zhang S., Geng S., Shi Y., Ma H., Liu B. (2022). Fabrication and characterization of Pickering high internal phase emulsions stabilized by Tartary buckwheat bran flour. Food Chem. X.

[124] Gutiérrez Á.L., Rico D., Ronda F., Martín-Diana A.B., Caballero P.A. (2022). Development of a gluten-free whole grain flour by combining soaking and high hydrostatic pressure treatments for enhancing functional, nutritional and bioactive properties. J. Cereal Sci..

[125] Salejda A.M., Olender K., Zielińska-Dawidziak M., Mazur M., Szperlik J., Miedzianka J., Zawiślak I., Kolniak-Ostek J., Szmaja A. (2022). Frankfurter-type sausage enriched with buckwheat by-product as a source of bioactive compounds. Foods.

[126] Atambayeva Z., Nurgazezova A., Assirzhanova Z., Urazbayev Z., Kambarova A., Dautova A., Kaygusuz M. (2023). Nutritional, physicochemical, textural and sensory characterization of horsemeat patties as affected by whole germinated green buckwheat and its flour. Int. J. Food Prop..

[127] Uzakov Y., Kaldarbekova M., Kuznetsova O. (2020). Improved technology for new-generation Kazakh national meat products. Foods Raw Mater..

[128] Sujka K., Cacak-Pietrzak G., Sułek A., Murgrabia K., Dziki D. (2022). Buckwheat hull-enriched pasta: Physicochemical and sensory properties. Molecules.

[129] Ghandehary Yazdi A.P., Kamali Rousta L., Azizi Tabrizzad M.H., Amini M., Tavakoli M., Yahyavi M. (2020). A review: New approach to enrich pasta with fruits and vegetables. Food Sci. Technol..

[130] Prasad C., Davis K.E., Imrhan V., Juma S., Vijayagopal P. (2019). Advanced glycation end products and risks for chronic diseases: Intervening through lifestyle modification. Am. J. Lifestyle Med..

[131] Semba R.D., Bandinelli S., Sun K., Guralnik J.M., Ferrucci L. (2009). Plasma carboxymethyl-lysine, an advanced glycation end product, and all-cause and cardiovascular disease mortality in older community-dwelling adults. J. Am. Geriatr. Soc..

[132] Abdulai I.L., Kwofie S.K., Gbewonyo W.S., Boison D., Puplampu J.B., Adinortey M.B. (2021). Multitargeted effects of vitexin and isovitexin on diabetes mellitus and its complications. Sci. World J..

[133] Brudzynski K., Miotto D. (2011). Honey melanoidins: Analysis of the compositions of the high molecular weight melanoidins exhibiting radical-scavenging activity. Food Chem..

[134] Qin P., Li W., Yang Y., Ren G. (2013). Changes in phytochemical compositions, antioxidant and α-glucosidase inhibitory activities during the processing of Tartary buckwheat tea. Food Res. Int..

[135] Yang X., Zhou Y., Wang B., Wang F., Han P., Li L. (2019). Tartary buckwheat extract and chitosan-coated tilapia (*Oreochromis niloticus*) fillets determine their shelf life. J. Food Sci..

[136] Matseychik I.V., Korpacheva S.M., Lomovsky I.O., Serasutdinova K.R. (2021). Influence of buckwheat by-products on the antiox-idant activity of functional desserts. IOP Conf. Ser. Earth Environ. Sci..

[137] Chen F., Xu Y., Ma K., Zhang Y., Jin T. (2025). Buckwheat allergy. Asian Pac. J. Allergy Immunol..

[138] Kerienė I., Mankevičienė A., Bliznikas S., Cesnuleviciene R., Janaviciene S., Jablonskytė-Raščė D., Maiksteniene S. (2016). The effect of buckwheat groats processing on the content of mycotoxins and phenolic compounds. CyTA J. Food.

[139] Krysińska-Traczyk E., Perkowski J., Dutkiewicz J. (2007). Levels of fungi and mycotoxins in the samples of grain and grain dust collected from five various cereal crops in Eastern Poland. Ann. Agric. Environ. Med..

[140] Sacco C., Donato R., Zanella B., Pini G., Pettini L., Marino M.F., Rookmin A.D., Marvasi M. (2020). Mycotoxins and flours: Effect of type of crop, organic production, packaging type on the recovery of fungal genus and mycotoxins. Int. J. Food Microbiol..

[141] Sugita-Konishi Y., Nakajima M., Tabata S., Ishikuro E., Tanaka T., Norizuki H., Itoh Y., Aoyama K., Fujita K., Kai S. (2006). Occurrence of aflatoxins, ochratoxin A, and fumonisins in retail foods in Japan. J. Food Prot..

[142] Norbäck D., Wieslander G. (2021). A review on epidemiological and clinical studies on buckwheat allergy. Plants.

[143] Heffler E., Pizzimenti S., Badiu I., Guida G., Rolla G. (2014). Buckwheat allergy: An emerging clinical problem in Europe. J. Allergy Ther..

[144] Škrabanja V., Kreft I., Golob T., Modic S., Ikeda M., Ikeda K., Kreft S., Bonafaccia G., Knapp M., Kosmelj K. (2004). Nutrient content in buckwheat milling fractions. Cereal Chem..

[145] Farooq M., Wani S., Mir S., Naseem Z. (2023). An overview of buckwheat allergy: A rare allergenic food. J. Food Compos. Anal..

[146] Kasar C., Thanushree M.P., Gupta S., Inamdar A.A. (2021). Milled fractions of common buckwheat (*Fagopyrum esculentum*) from the Himalayan regions: Grain characteristics, functional properties and nutrient composition. J. Food Sci. Technol..

[147] Zamaratskaia G., Gerhardt K., Knicky M., Wendin K. (2024). Buckwheat: An underutilized crop with attractive sensory qualities and health benefits. Crit. Rev. Food Sci. Nutr..

[148] Kreft I., Germ M., Golob A., Vombergar B., Bonafaccia F., Luthar Z. (2022). Impact of rutin and other phenolic substances on the digestibility of buckwheat grain metabolites. Int. J. Mol. Sci..

[149] European Parliament, Council of the European Union (2011). Regulation (EU) No 1169/2011 of the European Parliament and of the Council of 25 October 2011 on the provision of food information to consumers. Off. J. Eur. Union.

[150] U.S. Food and Drug Administration (FDA) Food Allergies. https://www.fda.gov/food/nutrition-food-labeling-and-critical-foods/food-allergies.

[151] Consumer Affairs Agency, Government of Japan Japan’s Food Labelling System. https://www.caa.go.jp/en/policy/food_labeling/assets/food_labeling_cms204_240425_01.pdf.

[152] European Commission (2023). Commission Regulation (EU) 2023/915 of 25 April 2023 on maximum levels for certain contaminants in food and repealing Regulation (EC) No 1881/2006. Off. J. Eur. Union.

[153] European Commission Novel Food Status Catalogue. https://food.ec.europa.eu/food-safety/novel-food/novel-food-status-catalogue_en.

